# Supplementary material for: Knowledge, attitudes and experiences of self-harm and suicide in low-income and middle-income countries: protocol for a systematic review
Source: BMJ Open. 2021 Jun 22;11(6):e041645. doi: 10.1136/bmjopen-2020-041645 (PMC8220466; doi:10.1136/bmjopen-2020-041645)
Supplement: Supplementary data [file bmjopen-2020-041645supp002.pdf]

Appendix 1: Knowledge, attitudes, and experiences of self-harm and suicide in low and middle income countries: Medline search strategy

Self-harm and suicide terms

1 Suicide/
2 Suicide.mp
3 Suicide, Attempted/
4 Suicide, Attempted.mp
5 Self-Injurious Behavior/
6 Self-Injurious Behavior.mp
7 Self-Mutilation/
8 Self-Mutilation.mp
9 Suicidal Ideation/
10 Suicidal Ideation.mp
11 Attempted Suicide/
12 Attempted Suicide.mp
13 Drug Overdose/
14 Drug Overdose.mp
15 ((Self adj2 cut$) or (para adj suicid$) or (attempt$ adj suicide$) or (suicid$ adj behavio$)).mp.
16 (auto?mutilat$ or cutt$ or over?dos$ or self?destruct$ or self?harm$ or self?immolat$ or self?inflict$ or self?injur$ or self?mutilat$ or self?poison$ or suicide$ or self?burn$).mp.
17 Or/1-16

Stakeholder terms

18 exp Health Personnel/
19 Health Personnel.mp
20 exp PHYSICIANS/
21 PHYSICIANS.mp
22 exp Personnel, Hospital/
23 Personnel, Hospital.mp
24 Social Workers/
25 Social Workers.mp
26 ((doctor$ or physician$ or hosp$ or medic$ or health?care or social or wel?fare) adj (profession$ or staff$ or officer$ or person$ or worker$)).mp
27 Family/
28 Famil$.mp
29 Friends/
30 Friend$.mp
31 Caregivers/
32 Caregiver$.mp
33 Criminals/
34 Criminal$.mp
35 Prisoners/
36 Prison$.mp
37 Social Justice/
38 Soci$ adj Just$.mp
39 (partner$ or parent$ or grandparent$ or children or sibling$ or famil$ or friend$ or relative$).mp.
40 (communit$ or societ$ or government$).mp.
41 (criminal$ or offender$ or prisoner$ or in?mate$).mp.
42 (justice adj system$).mp.

43 or/18-42

Knowledge and attitude terms

44 Attitude/
45 Attitude of Health Personnel/
46 Attitude to Death/
47 Knowledge/
48 Health Knowledge, Attitudes, Practice/
49 Awareness/
50 Education/
51 Health Education/
52 ((train$ or trainer$ or experienc$ or perspectiv$ or knowledg$ or attitud$ or awaren$ or educat$) adj health).mp.
53 social behavior/ or help-seeking behavior/ or self-control/ or shyness/ or social adjustment/ or social isolation/ or social marginalization/ or social skills/ or social stigma/ or social exclusion/ or social inclusion
54 Prejudice/
55 Taboo/
56 exp Shame/
57 (tabo$ or sham$ or stigma$ or prejudice$ or help?seek$ behavior$ or self?control$ or shy$ or (social adj adjust$ or behavio$ or isolate$ or margin$ or Stigm$ or skil$)).mp
58 or/44-57

LMIC terms

59 Developing Countries/
60 "low and middle income countr$".ab,ti.
61 LMIC.mp.
62 india/ or sikkim/ or pakistan/
63 exp Asia/
64 or/59-63

Self-harm and suicide, stakeholders, knowledge and attitude and LMIC combined (with limits)

65 17 and 43 and 58 and 64
66 limit 65 to humans
67 limit 66 (adolescent <13 to 17 years> or adult <18 to 64 years> or aged <65+ years>) (aged or "aged, 80 and over" or "frail elderly" or "middle aged" or "young adult" or adults or "teenager" or adolescent).mp
