# Supplementary material for: Knowledge, attitudes and experiences of self-harm and suicide in low-income and middle-income countries: protocol for a systematic review
Source: BMJ Open. 2021 Jun 22;11(6):e041645. doi: 10.1136/bmjopen-2020-041645 (PMC8220466; doi:10.1136/bmjopen-2020-041645)
Supplement: Supplementary data [file bmjopen-2020-041645supp003.pdf]

Appendix 2: Knowledge, attitudes, and experiences of self-harm and suicide in low and middle income countries systematic review: data extraction form

Notes for reviewers

Record any missing information as unclear or not described to ensure it is clear that the information was not found in the paper, not that you forgot to extract it

General information		
Date form completed		
Reviewer extracting data		
Study title		
Study authors		
Journal		
Year of publication		
Study author contact details		
Notes		
**Study eligibility for inclusion in review**		
Main focus on stakeholders' knowledge, attitudes and experiences of self-harm and/or suicide (excluding euthanasia or terrorism)	*Yes/No*	*Location in text (pg. #)*
Study population aged 16 and above (or can data from those aged 16 and above only can be extracted?)	*Yes/No*	*Location in text (pg. #)*
Low-middle income country	*Yes/No*	*Location in text (pg. #)*
Include or exclude	*Include/Exclude*	
Reason for exclusion	*Reasons for exclusion*<br>*1) Knowledge, attitudes and experience of self-harm/suicide not main concern of study (including terrorism and euthanasia) include main phenomenon being studied in notes – to be reviewed after 25 studies*<br>*1a) Completely irrelevant topic e.g. paper on depression, no mention of self-harm/suicide 1b) Focus on prevalence of suicide/self-harm*<br>*1c) Focus on risk factors of suicide/self-harm*<br>*1d) Focus on intervention only*<br>*1e) Mention of self-harm/suicide however topic not relevant to attitudes, knowledge and experiences of self-harm/suicide*<br>*2) Research not conducted in LMICs*<br>*3) Research population not 16 and over*<br>*4) Literature review*<br>*5) Commentary, book review, editorial*	
Notes		
**Characteristics of included studies: Participants**		

	Description as stated in paper	*Location in text (pg. #)*
Study location (Country and state/city/area) e.g. India, Bangalore		
Study setting e.g. hospital, community		
Study population e.g. nurses, community members		
Informed consent obtained	*Yes, No, Unclear*	
Total number of participants		
Exclusions and withdrawals		
Participant demographics e.g.<br>Age<br>Sex<br>Race/Ethnicity<br>Religious beliefs<br>Mental illness diagnosis<br>Physical illness diagnosis<br>Other demographics		
Notes		
**Characteristics of included studies: Methods**		
	Description as stated in paper	*Location in text (pg. #)*
Aim of study/Research question(s) (implicit or explicit in text?)		
Study methodology (or methodologies)		
Quantitative measures used e.g. Acceptability of Suicide Scale and information on whether measure is validated (if applicable)		
Quantitative analysis methods and procedure		
Qualitative methods used e.g. focus group, one-to-one interviews, vignettes (if applicable)		
Theoretical/epistemological perspectives underpinning qualitative research (explicit or reviewer's interpretation)		
Qualitative data analysis methods and procedure		
Start date and end date		
Notes		
**Characteristics of included studies: Results**		
	Description as stated in paper	*Location in text (pg. #)*
Qualitative results – direct quotes from participants (first order)		

Qualitative results – study author's interpretations of data (second order)		
Quantitative results		
Indicators of acceptability to users (if applicable)		
Suggested mechanisms of intervention action (if applicable)		
**Characteristics of included studies: Other information**		
	Description as stated in paper	*Location in text (pg. #)*
Key conclusions of authors		
References to other relevant studies		
Correspondence required by reviewers for further information (who, when, what requested)		
Notes		
