# Supplementary material for: Knowledge, attitudes and experiences of self-harm and suicide in low-income and middle-income countries: protocol for a systematic review
Source: BMJ Open. 2021 Jun 22;11(6):e041645. doi: 10.1136/bmjopen-2020-041645 (PMC8220466; doi:10.1136/bmjopen-2020-041645)
Supplement: Author's manuscript [file bmjopen-2020-041645.draft_revisions.pdf]

BMJ Open

BMJ Open is committed to open peer review. As part of this commitment we make the peer review history of every article we publish publicly available.

When an article is published we post the peer reviewers' comments and the authors' responses online. We also post the versions of the paper that were used during peer review. These are the versions that the peer review comments apply to.

The versions of the paper that follow are the versions that were submitted during the peer review process. They are not the versions of record or the final published versions. They should not be cited or distributed as the published version of this manuscript.

BMJ Open is an open access journal and the full, final, typeset and author-corrected version of record of the manuscript is available on our site with no access controls, subscription charges or pay-per-view fees (http://bmjopen.bmj.com).

If you have any questions on BMJ Open's open peer review process please email info.bmjopen@bmj.com

BMJ Open

Knowledge, attitudes, and experiences of self-harm and suicide in low and middle income countries: protocol for a systematic review

Journal:	*BMJ Open*
Manuscript ID	bmjopen-2020-041645
Article Type:	Protocol
Date Submitted by the Author:	13-Jun-2020

Complete List of Authors:	McPhillips, Rebecca; University of Manchester, Oxford Road, M13 9PL, Social Care and Society<br>Nafees, Sadia; Bangor University, North Wales Centre for Primary Care Research<br>Elahi, Anam ; The University of Manchester, Social Care and Society<br>Batool, Saqba; The University of Manchester, Social Care and Society<br>Krishna, Murali; Foundation for Research and Advocacy in Mental Health (FRAMe)<br>Krayer, Anne; Bangor University<br>Huxley, Peter; Bangor University<br>Chaudhry, Nasim ; Pakistan Institute of Living and Learning<br>Robinson, Catherine; The University of Manchester

Keywords:	Suicide & self-harm < PSYCHIATRY, PUBLIC HEALTH, MENTAL HEALTH

SCHOLARONE™
Manuscripts

I, the Submitting Author has the right to grant and does grant on behalf of all authors of the Work (as defined in the below author licence), an exclusive licence and/or a non-exclusive licence for contributions from authors who are: i) UK Crown employees; ii) where BMJ has agreed a CC-BY licence shall apply, and/or iii) in accordance with the terms applicable for US Federal Government officers or employees acting as part of their official duties; on a worldwide, perpetual, irrevocable, royalty-free basis to BMJ Publishing Group Ltd ("BMJ") its licensees and where the relevant Journal is co-owned by BMJ to the co-owners of the Journal, to publish the Work in this journal and any other BMJ products and to exploit all rights, as set out in our licence.

The Submitting Author accepts and understands that any supply made under these terms is made by BMJ to the Submitting Author unless you are acting as an employee on behalf of your employer or a postgraduate student of an affiliated institution which is paying any applicable article publishing charge ("APC") for Open Access articles. Where the Submitting Author wishes to make the Work available on an Open Access basis (and intends to pay the relevant APC), the terms of reuse of such Open Access shall be governed by a Creative Commons licence – details of these licences and which Creative Commons licence will apply to this Work are set out in our licence referred to above.

Other than as permitted in any relevant BMJ Author's Self Archiving Policies, I confirm this Work has not been accepted for publication elsewhere, is not being considered for publication elsewhere and does not duplicate material already published. I confirm all authors consent to publication of this Work and authorise the granting of this licence.

For peer review only

Knowledge, attitudes, and experiences of self-harm and suicide in low and middle income countries: protocol for a systematic review

Rebecca McPhillips*[1]

Sadia Nafees[2]

Anam Elahi[1]

Saqba Batool[1]

Murali Krishna[2]

Anne Krayer[2]

Peter Huxley[2]

Nasim Chaudhry[3],

Catherine Robinson[1]

*Room C4.5, Ellen Wilkinson Building, Oxford Road, Manchester, M13 9PL.

Rebecca.mcphillips@manchester.ac.uk +44(0)161 275 5678

[1] Social Care and Society (SCS), Division of Population Health, Health Services Research and Primary Care, School of Health Sciences, Faculty of Biology, Medicine and Health, University of Manchester, Manchester, UK.

[2] Centre For Mental Health And Society, Bangor University, Wales.

[3] Chief Executive Officer, Pakistan Institute of Living and Learning, Karachi, Pakistan

Word count: 2,022

For peer review only

ABSTRACT

Introduction: Over 800,000 people die due to suicide each year and suicide presents huge psychological, economic and social burdens for individuals, communities and countries as a whole. Low and middle income countries (LMICs) are disproportionately affected by suicide. The strongest risk factor for suicide is a previous suicide attempt, and other types of self-harm have been found to be robust predictors of suicidal behaviour. An approach that brings together multiple sectors including education, labour, business, law, politics and the media is crucial to tackling suicide and self-harm. The World Health Organization highlights that evaluations of the knowledge and attitudes that priority groups, not only healthcare staff, have of mental health and suicidal behaviour are key to suicide prevention strategies. The aim of this systematic review is to examine the knowledge, attitudes and experiences different stakeholders in LMICs have of self-harm and suicide.

Methods and analysis: Medline, Embase, PsycInfo, CINAHL, BNI, Social Sciences and Cochrane library will be searched. Reviewers working independently of each other will screen search results, select studies for inclusion, extract and check extracted data, and rate the quality of the studies using the STROBE and CASP checklists. In anticipation of heterogeneity, a narrative synthesis of quantitative studies will be provided and meta-ethnography will be used to synthesise qualitative studies.

Ethics and dissemination*:* Ethical approval is not required. A report will be provided for the funding body, and the systematic review will be submitted for publication in a high-impact, peer-reviewed, open access journal. Results will also be disseminated at conferences, seminars, congresses and symposia and to relevant stakeholders.

PROSPERO registration number: CRD42019135323

Strengths and limitations of this study

- This systematic review protocol has been written according to the Preferred Reporting Items for Systematic review and Meta-Analysis (PRISMA-P) 2015 checklist.

For peer review only - http://bmjopen.bmj.com/site/about/guidelines.xhtml

- The review will conform to the PRISMA statement and to the Cochrane systematic review literature guidelines when results are reported
- A strength of this review is the mixed methods approach, which is particularly suited to the investigation of complex topics
- A limitation of the review is the inclusion of peer reviewed studies only, however language restrictions will not be applied
- The findings from this review will be form a robust basis for the development of a community survey on knowledge and attitudes towards self-harm and suicide in South Asia.

Keywords

Self-harm; suicide; attitudes; knowledge; experience; quantitative; qualitative; mixed methods, low and middle income countries, LMICs

INTRODUCTION

The World Health Organization (WHO) estimate that over 800,000 people die due to suicide each year; 1 person every 40 seconds [1]. Suicide disproportionately affects low and middle income countries (LMICs), in 2014 the WHO reported that 75.5% of suicides globally occur in LMICs, and in South East Asia suicide is the leading cause of death in 15-29 year olds [1]. However, the under-reporting and misclassification of suicide as a cause of death in LMICs mean that suicide rates are likely higher than reported [1, 2]. Every suicide death is a tragedy for families, friends and communities and suicide presents huge psychological, economic and social burdens for individuals, communities and countries as a whole [1].

The strongest risk factor for suicide is a previous suicide attempt, and the WHO suggest that for each adult that dies from suicide there may be 20 more suicide attempts [1]. Suicide attempts and suicide are types of self-harm that are distinct from non-suicidal self-injury (NSSI) in terms of intent, however NSSI has also been found to be a robust predictor of suicidal behaviour [3, 4, 5]. A review of

For peer review only - http://bmjopen.bmj.com/site/about/guidelines.xhtml

the literature on the links between NSSI and suicidal behaviour found that people who engaged in NSSI were significantly more likely to report suicidal ideation and to have attempted suicide than those who did not [3]. Furthermore, the link between NSSI and suicidal behaviour remained after controlling for age, gender, and ethnicity and NSSI was a stronger predictor of suicidal behaviour than depression, hopelessness, post-traumatic stress, borderline personality disorder, family functioning and child abuse [3].

Suicide and self-harm are the result of complex interactions between genetic, psychological, biological, cultural, sociodemographic and social factors [1, 6, 7]. Although the healthcare sector clearly has a vital role to play in tackling suicide and self-harm, an approach that brings together multiple sectors including education, labour, business, law, politics and the media is crucial [1, 8].

The knowledge, attitudes and experiences stakeholders from various sectors have of suicide and self-harm are likely to influence suicide and self-harm prevention and intervention strategies. A recent review by the WHO [8] highlights that evaluations of the knowledge and attitudes that priority groups, for example policy makers and community groups, not only healthcare staff, have of mental health and suicidal behaviour are key to the collection of high quality surveillance data and prevention strategies. Reviews to date have focused on the knowledge, attitudes and experiences that healthcare professionals have towards self-harm and suicide [9, 10, 11, 12]. The aim of this systematic review is to examine various stakeholders' knowledge, attitudes and experiences of self-harm and suicide. This systematic review is being undertaken as part of the South Asia Self Harm Initiative (SASHI) project, which aims to help to find effective responses to self-harm and suicide in South Asia by building capability and capacity in research infrastructure and expertise in the region. Findings from this systematic review will be used to inform the development of a survey on knowledge, attitudes and well-being in South Asia. Thus, we are particularly interested in studies conducted in South Asia and countries with comparable healthcare systems or cultural backgrounds.

Research question

For peer review only - http://bmjopen.bmj.com/site/about/guidelines.xhtml

For peer review only

The SPICE (**S**etting, **P**erspective, phenomena of **I**nterest, **C**omparison, **E**valuation) framework was used to generate the research question that will be addressed by this systematic review [13]:

- What are stakeholders' knowledge, attitudes and experiences of self-harm and suicide in LMICs?

METHODS AND ANALYSIS

This protocol conforms to the Preferred Reporting Items for Systematic Reviews and Meta-Analyses Protocols (PRISMA-P) checklist (see Supplementary File 1) [14]. We will conform to the PRISMA statement and to the Cochrane systematic review literature guidelines when reporting the results [15, 16]. This systematic review has been registered on PROSPERO [17].

Search strategy

A Betsi Cadwaladr University Health Board (BCUHB) librarian with expertise in systematic reviews has assisted the authors in the development of the search strategy (see Appendix 1). We will search Medline, Embase, PsycInfo, CINAHL, BNI, Social Sciences and Cochrane library. We will not apply any language restrictions to the search criteria. EndNote and Microsoft Word will be used to manage initial search results, screening and data throughout the review. We will update the searches prior to publication to ensure the latest papers are included. Reference lists from included studies and any identified systematic or literature reviews will also be searched by hand. Study authors will be contacted in instances when it has not been possible to retrieve full text articles and when clarification regarding inclusion criteria e.g. participant age, is required.

Study selection criteria

Inclusion criteria are empirical studies conducted in LMICs, as defined by the Organisation for Economic Co-operation and Development [18], irrespective of the study design, whose focus is on the knowledge, attitudes or experiences of stakeholders towards self-harm and/or suicide, where participants are aged 16 years and above. Stakeholders are people who have experienced self-harm

For peer review only - http://bmjopen.bmj.com/site/about/guidelines.xhtml

and/or have attempted suicide themselves, relatives, friends, co-workers, and healthcare workers of those who have self-harmed, attempted or committed suicide and people in the social, healthcare, government, and criminal justice sectors. Exclusion criteria are studies conducted in high income countries (HICs) and studies whose participants are not aged 16 years and above. Studies whose main focus is on the prevalence and/or predictors of self-harm and/or suicide, relationships between state and/or trait characteristics and self-harm and/or suicide, euthanasia, terrorism, or epidemiology will also be excluded. Systematic and literature reviews will be consulted for relevant references but will not be included in the review. Opinion pieces, editorials, book reviews, and conference and poster abstracts will not be included in the review.

The selection of studies for inclusion will adhere to the Cochrane guidelines and the process of selection of eligible studies will be illustrated via a PRISMA diagram [16]. Following deduplication of search results in EndNote, the following screening process will be undertaken in order to select studies for inclusion in the systematic review:

1) Titles and abstracts will be read by two reviewers independently, and relevance and fit with the inclusion criteria will be assessed. Those of no obvious relevance will be excluded and any disagreements will be resolved with a third reviewer (and the wider expert group if necessary).

2) Full text articles of remaining studies will be retrieved and read by two reviewers independently to assess their suitability for inclusion in the final review, disagreements will be resolved by discussion with a third reviewer (and the wider expert group if necessary). Both reviewers will populate a piloted pro-forma for each full text paper read (see Appendix 2).

Data extraction

For peer review only - http://bmjopen.bmj.com/site/about/guidelines.xhtml

Data will be extracted from selected studies by one reviewer, and a second reviewer will check for accuracy. Extracted data will be recorded on a piloted pro-forma (see Appendix 2), and will reflect the inclusion criteria and the designated aims of the review, derived from the article as a whole. Discrepancies will be resolved through discussion (with the wider expert group if necessary). Additional data will be requested from study authors when necessary. Data extraction of qualitative studies (and for qualitative components in studies with mixed methods) will adhere to the same methods and will be reviewed independently.

Outcomes

Outcomes of interest include:

- The identification of relevant information on stakeholders' knowledge, attitudes and experiences of self-harm and suicide, particularly in South Asia and in countries with comparable healthcare systems and cultural backgrounds
- The quantitative methods and measures that have been used to investigate stakeholders' attitudes towards and knowledge about self-harm and suicide and their psychometric properties
- The qualitative methods that have been used to investigate stakeholders' attitudes towards, knowledge about, and experiences of self-harm and suicide.

The identified outcomes will inform the development of a survey on knowledge, attitudes and well-being in South Asia as part of the SASHI project.

Quality assessment

All eligible studies will be subject to quality appraisal. The quality of included quantitative studies will be appraised using the STROBE checklist [19]. The STROBE Statement consists of a checklist of 22 items, which relate to the title, abstract, introduction, methods, results and discussion sections of articles. Eighteen items are common to cohort studies, case-control studies and cross-sectional studies and four are specific to each of the three study designs. The quality of included qualitative studies will

For peer review only - http://bmjopen.bmj.com/site/about/guidelines.xhtml

be appraised using the CASP checklist [20]. The 10-item CASP tool was considered to be the most suitable tool to consider the quality parameters of qualitative work, and is a well-validated and accepted tool [15]. Both the STROBE and CASP checklists will be applied independently by two reviewers and any disagreements will be resolved with a third reviewer (and the wider expert group if necessary).

Studies will not be excluded on the basis of poor quality alone, rather we will discuss the implications of including studies rated as being of poor quality and place them within context of the wider literature. This low threshold for inclusion will be applied so that the review can benefit from researcher insight and theoretical as well as empirical contributions. The relative quality of included studies will be critically considered during the analysis and in reference to the developed synthesis and we will attempt to assess the trustworthiness of the evidence in terms of transparency in reporting, consensus and expertise of the team and relevant stakeholders, and draw on common sense and expertise as well as evidence.

Descriptive analysis and data synthesis

We anticipate that the quantitative studies included in the review will be heterogenous and this will prevent meta-analysis. We will provide a narrative synthesis of quantitative studies, structured around population characteristics and the geographical region of studies. We will provide summaries of the quantitative methods and measures used to investigate stakeholders' attitudes towards and knowledge about self-harm and suicide and their psychometric properties.

Meta-ethnography will be used to synthesise qualitative studies [21]. Initially reciprocal translation will be performed by comparing the concepts presented in different studies. A chronological approach will be taken to reciprocal translation; studies will be arranged chronologically, concepts from papers one and two will be compared, and the synthesis of papers one and two will then be compared with paper three, and so forth, as is described elsewhere [22]. When contradictions between studies are identified, we will perform refutational synthesis by exploring and explaining these. A 'lines-of-

For peer review only - http://bmjopen.bmj.com/site/about/guidelines.xhtml

argument' synthesis, that links and explains concepts presented by different studies, will be conducted so that an interpretation of all included studies can be presented.

Two reviewers will lead data synthesis. Emergent analysis, and any discrepancies, will be discussed with other members of the review team. Microsoft Office software will be used to facilitate data synthesis.

Patient and public involvement

No patients or members of the public were involved in the design of this study.

Amendments

An amendment has been made to the initial registration of this systematic review in PROSPERO, which details that only studies from LMICs will be included in this review, and studies from HICs will be excluded from this review. Any further amendments to this protocol will be documented in the full review.

ETHICS AND DISSEMINATION

Ethics approval is not required as this is a protocol for the systematic review of previously published data. In addition to a report to the funding body, we intend to submit the systematic review for publication in a high-impact, peer-reviewed journal. We will select an open access journal to ensure free access to undergraduate and graduate students, researchers, academics and research groups. Results will also be disseminated at conferences seminars, congresses and symposia and to relevant stakeholders.

Acknowledgements: The authors would like to thank Mrs Nia Morris (NM) from BCUHB library for her contribution to the development of the search strategy.

For peer review only - http://bmjopen.bmj.com/site/about/guidelines.xhtml

Contributions: CR, RM, PH, AK, MK conceived the research idea. SN led the development of the search strategy, with input from NM, RM, AK, PH. RM, SN, MK, AK, PH, CR conceived the manuscript. RM lead the drafting of the manuscript with contributions from SN, AE, SB, MK, AK, PH, NC, CR. All authors approved the final manuscript.

Funding: This manuscript was funded by UK Research and Innovation Global Challenge Research Fund (ref: MR/P028144/1). The finder did not have any role in developing this protocol.

Competing interests: None declared

For peer review only

References

[1] World Health Organization. Preventing suicide: a global imperative. World Health Organization, 2014. Available: https://apps.who.int/iris/bitstream/handle/10665/131056/9789241564779_eng.pdf;jsessionid=F6255CAAD6496DA078DE41FAB0C5CCBF?sequence=1 . [Accessed 04 May 2020].

[2] Jordans MJD, Kaufman A, Brenman NF, *et al*. Suicide in South Asia: a scoping review. *BMC Psychiatry* 2014;**14**:358.doi:10.1186/s12888-014-0358-9 pmid: https://www.ncbi.nlm.nih.gov/pubmed/25539951

[3] Hamza CA, Stewart SL, Willoughby T. Examining the link between nonsuicidal self-injury and suicidal behaviour: a review of the literature and integrated model. *Clin Psychol Rev* 2012;**6** 482-95.doi:10.1016/j.cpr.2012.05.003. pmid: https://www.ncbi.nlm.nih.gov/pubmed/22717336

[4] Nock MK. Self-injury. *Annu Rev Clin Psychol* 2010;**6**:339-63. doi: 10.1146/annurev.clinpsy.121208.131258 pmid: https://www.ncbi.nlm.nih.gov/pubmed/20192787

[5] Owens D, Horrocks J, House A. Fatal and non-fatal repetition of self-harm: systematic review. *Br J Psychiatry* 2002;**181**:193-99. doi: 10.1192/bjp.181.3.193 pmid: https://www.ncbi.nlm.nih.gov/pubmed/12204922

[6] Hawton K, Sanders KEA, O'Connor RC. Self-harm and suicide in adolescents. *Lancet* 2012;**379**:2373-82. doi: 10.1016/S0140-6736(12)60322-5 pmid: https://www.ncbi.nlm.nih.gov/pubmed/22726518

[7] Yip PSF, Caine E, Yousef S, *et al*. Means restriction for suicide prevention. *Lancet* 2012;379:2393-2399. doi: 10.1016/S0140-6736(12)60521-2

[8] World Health Organization. National suicide prevention strategies: progress, examples and indicators. World Health Organization, 2018. Available: https://apps.who.int/iris/bitstream/handle/10665/279765/9789241515016-eng.pdf?ua=1 . [accessed 04 May 2020].

[9] Karman P, Kool N, Poslawsky IE, *et al.* Nurses' attitudes towards self-harm: a literature review. *J Psychiatr Ment Health Nurs* 2015;**22**:65-75. doi: 10.1111/jpm.12171 pmid: https://www.ncbi.nlm.nih.gov/pubmed/25490929

[10] O'Connor S, Glover L. Hospital staff experiences of their relationships with adults who self-harm: a meta-synthesis. *Psychol Psychother* 2017;**90**:480-501. doi: 10.1111/papt.12113. pmid: https://www.ncbi.nlm.nih.gov/pubmed/28035740

[11] Rees N, Rapport F, Thomas G, *et al*. Perceptions of paramedic and emergency care workers of those who self-harm: a systematic review of the quantitative literature. *J Psychosom Res*

For peer review only - http://bmjopen.bmj.com/site/about/guidelines.xhtml

2014:**77**;449-456. doi: 10.1016/j.jpsychores.2014.09.006. pmid: https://www.ncbi.nlm.nih.gov/pubmed/25263398

[12] Saunders KEA, Hawton K, Fortune S, *et al*. Attitudes and knowledge of clinical staff regarding people who self-harm: a systematic review. *J Affect Disord* 2012;**139**:205-216. doi: 10.1016/j.jad.2011.08.024 pmid: https://www.ncbi.nlm.nih.gov/pubmed/21925740

[13] Booth A. Clear and present questions: formulating questions for evidence based practice. *Library Hi Tech* 2006;**24**:355-368. doi: 10.1108/07378830610692127

[14] Shamseer L, Moher D, Clarke M, *et al*. Preferred reporting items for systematic review and meta-analysis protocols (PRISMA-P) 2015: elaboration and explanation. *BMJ* 2015;**350**:g7647. doi: 10.1136/bmj.g7647 pmid: https://www.ncbi.nlm.nih.gov/pubmed/25555855

[15] Higgins JPT, Green S (editors). Cochrane handbook for systematic reviews of interventions Version 6. The Cochrane Collaboration, 2019. Available: http://handbook.cochrane.org. [Accessed 04 May 2020].

[16] Liberati A, Altman DG, Tetzlaff J, *et al*. The PRISAM statement for reporting systematic reviews and meta-analyses of studies that evaluate healthcare interventions: explanation and elaboration. *BMJ* 2009;**339**:b2700. doi: 10.1136/bmj.b2700 pmid: https://www.ncbi.nlm.nih.gov/pubmed/19622552

[17] McPhillips R, Nafees S, Krishna M, *et al*. Knowledge, attitudes, and experiences of self-harm and suicide in low and middle income countries: a systematic review. PROSPERO 2019 CRD42019135323. Available: https://www.crd.york.ac.uk/prospero/display_record.php?ID=CRD42019135323 [Accessed 04 May 2020]

For peer review only - http://bmjopen.bmj.com/site/about/guidelines.xhtml

[18] Organisation for Economic Co-operation and Development. DAC List of ODA recipients for reporting on aid in 2018 and 2019. Organisation for Economic Co-operation and Development, 2019. Available: http://www.oecd.org/dac/financing-sustainable-development/development-finance-standards/daclist.htm [Accessed 04 May 2020]

[19] von Elm E, Altman DG, Egger M, *et al.* STROBE Initiative. The strengthening the reporting in observational studies in epidemiology (STROBE) statement guidelines for reporting observational studies. Lancet 2007;**370**:1453-57 doi: 0.1016/S0140-6736(07)61602-X pmid: https://www.ncbi.nlm.nih.gov/pubmed/18064739

[20] Critical Appraisal Skills Programme. CASP qualitative checklist. Critical Appraisal Skills Programme , 2018. Available: https://casp-uk.net/wp-content/uploads/2018/01/CASP-Qualitative-Checklist-2018.pdf
[Accessed: 04 May 2020]

[21] Noblit GW, Hare RD. Meta-ethnography: synthesising qualitative studies. Sage Publications.

[22] Atkins S, Lewin S, Smith H *et al*. Conducting a meta-ethnography of qualitative literature: lessons learnt. *BMC Med Res Methodol* 2008;**8**:21. doi: 10.1186/1471-2288-8-21 pmid: https://www.ncbi.nlm.nih.gov/pubmed/ 18416812

For peer review only - http://bmjopen.bmj.com/site/about/guidelines.xhtml

For peer review only

For peer review only - http://bmjopen.bmj.com/site/about/guidelines.xhtml

For peer review only

Supplementary file 1: PRISMA-P (Preferred Reporting Items for Systematic review and Meta-Analysis Protocols) 2015 checklist: recommended items to address in a systematic review protocol*

Section and topic	Item No	Checklist item	Complete
**ADMINISTRATIVE INFORMATION**			
Title:			
Identification	1a	Identify the report as a protocol of a systematic review	X
Update	1b	If the protocol is for an update of a previous systematic review, identify as such	N/A
Registration	2	If registered, provide the name of the registry (such as PROSPERO) and registration number	X
Authors:			
Contact	3a	Provide name, institutional affiliation, e-mail address of all protocol authors; provide physical mailing address of corresponding author	X
Contributions	3b	Describe contributions of protocol authors and identify the guarantor of the review	X
Amendments	4	If the protocol represents an amendment of a previously completed or published protocol, identify as such and list changes; otherwise, state plan for documenting important protocol amendments	X
Support:			
Sources	5a	Indicate sources of financial or other support for the review	X
Sponsor	5b	Provide name for the review funder and/or sponsor	X
Role of sponsor or funder	5c	Describe roles of funder(s), sponsor(s), and/or institution(s), if any, in developing the protocol	X
**INTRODUCTION**			
Rationale	6	Describe the rationale for the review in the context of what is already known	X
Objectives	7	Provide an explicit statement of the question(s) the review will address with reference to participants, interventions, comparators, and outcomes (PICO)	X
**METHODS**			
Eligibility criteria	8	Specify the study characteristics (such as PICO, study design, setting, time frame) and report characteristics (such as years considered, language, publication	X

For peer review only

		status) to be used as criteria for eligibility for the review	
Information sources	9	Describe all intended information sources (such as electronic databases, contact with study authors, trial registers or other grey literature sources) with planned dates of coverage	X
Search strategy	10	Present draft of search strategy to be used for at least one electronic database, including planned limits, such that it could be repeated	X
Study records:			
Data management	11a	Describe the mechanism(s) that will be used to manage records and data throughout the review	X
Selection process	11b	State the process that will be used for selecting studies (such as two independent reviewers) through each phase of the review (that is, screening, eligibility and inclusion in meta-analysis)	X
Data collection process	11c	Describe planned method of extracting data from reports (such as piloting forms, done independently, in duplicate), any processes for obtaining and confirming data from investigator	X
Data items	12	List and define all variables for which data will be sought (such as PICO items, funding sources), any pre-planned data assumptions and simplifications	X
Outcomes and prioritization	13	List and define all outcomes for which data will be sought, including prioritization of main and additional outcomes, with rationale	X
Risk of bias in individual studies	14	Describe anticipated methods for assessing risk of bias of individual studies, including whether this will be done at the outcome or study level, or both; state how this information will be used in data synthesis	X
Data synthesis	15a	Describe criteria under which study data will be quantitatively synthesised	X
	15b	If data are appropriate for quantitative synthesis, describe planned summary measures, methods of handling data and methods of combining data from studies, including any planned exploration of consistency (such as I^2, Kendall's τ)	N/A
	15c	Describe any proposed additional analyses (such as sensitivity or subgroup analyses, meta-regression)	X
	15d	If quantitative synthesis is not appropriate, describe the type of summary planned	X
Meta-bias(es)	16	Specify any planned assessment of meta-bias(es) (such as publication bias across studies, selective reporting within studies)	N/A
Confidence in cumulative evidence	17	Describe how the strength of the body of evidence will be assessed (such as GRADE)	N/A

For peer review only

*** It is strongly recommended that this checklist be read in conjunction with the PRISMA-P Explanation and Elaboration (cite when available) for important clarification on the items. Amendments to a review protocol should be tracked and dated. The copyright for PRISMA-P (including checklist) is held by the PRISMA-P Group and is distributed under a Creative Commons Attribution Licence 4.0.**

From: Shamseer L, Moher D, Clarke M, Ghersi D, Liberati A, Petticrew M, Shekelle P, Stewart L, PRISMA-P Group. Preferred reporting items for systematic review and meta-analysis protocols (PRISMA-P) 2015: elaboration and explanation. BMJ. 2015 Jan 2;349(jan02 1):g7647.

For peer review only

Appendix 1: Knowledge, attitudes, and experiences of self-harm and suicide in low and middle income countries: Medline search strategy

Self-harm and suicide terms

1 Suicide/
2 Suicide.mp
3 Suicide, Attempted/
4 Suicide, Attempted.mp
5 Self-Injurious Behavior/
6 Self-Injurious Behavior.mp
7 Self-Mutilation/
8 Self-Mutilation.mp
9 Suicidal Ideation/
10 Suicidal Ideation.mp
11 Attempted Suicide/
12 Attempted Suicide.mp
13 Drug Overdose/
14 Drug Overdose.mp
15 ((Self adj2 cut$) or (para adj suicid$) or (attempt$ adj suicide$) or (suicid$ adj behavio$)).mp.
16 (auto?mutilat$ or cutt$ or over?dos$ or self?destruct$ or self?harm$ or self?immolat$ or self?inflict$ or self?injur$ or self?mutilat$ or self?poison$ or suicide$ or self?burn$).mp.
17 Or/1-16

Stakeholder terms

18 exp Health Personnel/
19 Health Personnel.mp
20 exp PHYSICIANS/
21 PHYSICIANS.mp
22 exp Personnel, Hospital/
23 Personnel, Hospital.mp
24 Social Workers/
25 Social Workers.mp
26 ((doctor$ or physician$ or hosp$ or medic$ or health?care or social or wel?fare) adj (profession$ or staff$ or officer$ or person$ or worker$)).mp
27 Family/
28 Famil$.mp
29 Friends/
30 Friend$.mp
31 Caregivers/
32 Caregiver$.mp
33 Criminals/
34 Criminal$.mp
35 Prisoners/
36 Prison$.mp
37 Social Justice/
38 Soci$ adj Just$.mp
39 (partner$ or parent$ or grandparent$ or children or sibling$ or famil$ or friend$ or relative$).mp.
40 (communit$ or societ$ or government$).mp.
41 (criminal$ or offender$ or prisoner$ or in?mate$).mp.
42 (justice adj system$).mp.

For peer review only

43 or/18-42

Knowledge and attitude terms

44 Attitude/
45 Attitude of Health Personnel/
46 Attitude to Death/
47 Knowledge/
48 Health Knowledge, Attitudes, Practice/
49 Awareness/
50 Education/
51 Health Education/
52 ((train$ or trainer$ or experienc$ or perspectiv$ or knowledg$ or attitud$ or awaren$ or educat$) adj health).mp.
53 social behavior/ or help-seeking behavior/ or self-control/ or shyness/ or social adjustment/ or social isolation/ or social marginalization/ or social skills/ or social stigma/ or social exclusion/ or social inclusion
54 Prejudice/
55 Taboo/
56 exp Shame/
57 (tabo$ or sham$ or stigma$ or prejudice$ or help?seek$ behavior$ or self?control$ or shy$ or (social adj adjust$ or behavio$ or isolate$ or margin$ or Stigm$ or skil$)).mp
58 or/44-57

LMIC terms

59 Developing Countries/
60 "low and middle income countr$".ab,ti.
61 LMIC.mp.
62 india/ or sikkim/ or pakistan/
63 exp Asia/
64 or/59-63

Self-harm and suicide, stakeholders, knowledge and attitude and LMIC combined (with limits)

65 17 and 43 and 58 and 64
66 limit 65 to humans
67 limit 66 (adolescent <13 to 17 years> or adult <18 to 64 years> or aged <65+ years>) (aged or "aged, 80 and over" or "frail elderly" or "middle aged" or "young adult" or adults or "teenager" or adolescent).mp

For peer review only

Appendix 2: Knowledge, attitudes, and experiences of self-harm and suicide in low and middle income countries systematic review: data extraction form

Notes for reviewers

Record any missing information as unclear or not described to ensure it is clear that the information was not found in the paper, not that you forgot to extract it

**General information**		
Date form completed		
Reviewer extracting data		
Study title		
Study authors		
Journal		
Year of publication		
Study author contact details		
Notes		
**Study eligibility for inclusion in review**		
Main focus on stakeholders' knowledge, attitudes and experiences of self-harm and/or suicide (excluding euthanasia or terrorism)	*Yes/No*	*Location in text (pg. #)*
Study population aged 16 and above (or can data from those aged 16 and above only can be extracted?)	*Yes/No*	*Location in text (pg. #)*
Low-middle income country	*Yes/No*	*Location in text (pg. #)*
Include or exclude	*Include/Exclude*	
Reason for exclusion	*Reasons for exclusion*<br>*1) Knowledge, attitudes and experience of self-harm/suicide not main concern of study (including terrorism and euthanasia) include main phenomenon being studied in notes – to be reviewed after 25 studies*<br>*1a) Completely irrelevant topic e.g. paper on depression, no mention of self-harm/suicide 1b) Focus on prevalence of suicide/self-harm*<br>*1c) Focus on risk factors of suicide/self-harm*<br>*1d) Focus on intervention only*<br>*1e) Mention of self-harm/suicide however topic not relevant to attitudes, knowledge and experiences of self-harm/suicide*<br>*2) Research not conducted in LMICs*<br>*3) Research population not 16 and over*<br>*4) Literature review*<br>*5) Commentary, book review, editorial*	
Notes		
**Characteristics of included studies: Participants**		

For peer review only

	Description as stated in paper	*Location in text (pg. #)*
Study location (Country and state/city/area) e.g. India, Bangalore		
Study setting e.g. hospital, community		
Study population e.g. nurses, community members		
Informed consent obtained	*Yes, No, Unclear*	
Total number of participants		
Exclusions and withdrawals		
Participant demographics e.g.<br>Age<br>Sex<br>Race/Ethnicity<br>Religious beliefs<br>Mental illness diagnosis<br>Physical illness diagnosis<br>Other demographics		
Notes		
**Characteristics of included studies: Methods**		
	Description as stated in paper	*Location in text (pg. #)*
Aim of study/Research question(s) (implicit or explicit in text?)		
Study methodology (or methodologies)		
Quantitative measures used e.g. Acceptability of Suicide Scale and information on whether measure is validated (if applicable)		
Quantitative analysis methods and procedure		
Qualitative methods used e.g. focus group, one-to-one interviews, vignettes (if applicable)		
Theoretical/epistemological perspectives underpinning qualitative research (explicit or reviewer's interpretation)		
Qualitative data analysis methods and procedure		
Start date and end date		
Notes		
**Characteristics of included studies: Results**		
	Description as stated in paper	*Location in text (pg. #)*
Qualitative results – direct quotes from participants (first order)		
Qualitative results – study		

For peer review only

author's interpretations of data (second order)		
Quantitative results		
Indicators of acceptability to users (if applicable)		
Suggested mechanisms of intervention action (if applicable)		
**Characteristics of included studies: Other information**		
	Description as stated in paper	*Location in text (pg. #)*
Key conclusions of authors		
References to other relevant studies		
Correspondence required by reviewers for further information (who, when, what requested)		
Notes		

For peer review only

BMJ Open

Knowledge, attitudes, and experiences of self-harm and suicide in low and middle income countries: protocol for a systematic review

Journal:	*BMJ Open*
Manuscript ID	bmjopen-2020-041645.R1
Article Type:	Protocol
Date Submitted by the Author:	09-Jan-2021
Complete List of Authors:	McPhillips, Rebecca; The University of Manchester, Social Care and Society<br>Nafees, Sadia; Bangor University, North Wales Centre for Primary Care Research<br>Elahi, Anam ; The University of Manchester, Social Care and Society<br>Batool, Saqba; The University of Manchester, Social Care and Society<br>Krishna, Murali; Bangor University<br>Krayer, Anne; Bangor University<br>Huxley, Peter; Bangor University<br>Robinson, Catherine; The University of Manchester
**Primary Subject Heading**:	Global health
Secondary Subject Heading:	Mental health
Keywords:	Suicide & self-harm < PSYCHIATRY, PUBLIC HEALTH, MENTAL HEALTH

SCHOLARONE™
Manuscripts

I, the Submitting Author has the right to grant and does grant on behalf of all authors of the Work (as defined in the below author licence), an exclusive licence and/or a non-exclusive licence for contributions from authors who are: i) UK Crown employees; ii) where BMJ has agreed a CC-BY licence shall apply, and/or iii) in accordance with the terms applicable for US Federal Government officers or employees acting as part of their official duties; on a worldwide, perpetual, irrevocable, royalty-free basis to BMJ Publishing Group Ltd ("BMJ") its licensees and where the relevant Journal is co-owned by BMJ to the co-owners of the Journal, to publish the Work in this journal and any other BMJ products and to exploit all rights, as set out in our licence.

The Submitting Author accepts and understands that any supply made under these terms is made by BMJ to the Submitting Author unless you are acting as an employee on behalf of your employer or a postgraduate student of an affiliated institution which is paying any applicable article publishing charge ("APC") for Open Access articles. Where the Submitting Author wishes to make the Work available on an Open Access basis (and intends to pay the relevant APC), the terms of reuse of such Open Access shall be governed by a Creative Commons licence – details of these licences and which Creative Commons licence will apply to this Work are set out in our licence referred to above.

Other than as permitted in any relevant BMJ Author's Self Archiving Policies, I confirm this Work has not been accepted for publication elsewhere, is not being considered for publication elsewhere and does not duplicate material already published. I confirm all authors consent to publication of this Work and authorise the granting of this licence.

For peer review only

Knowledge, attitudes, and experiences of self-harm and suicide in low and middle income countries: protocol for a systematic review

Rebecca McPhillips*[1]

Sadia Nafees[2]

Anam Elahi[1]

Saqba Batool[1]

Murali Krishna[2]

Anne Krayer[2]

Peter Huxley[2]

Nasim Chaudhry[3],

Catherine Robinson[1]

*Room C4.5, Ellen Wilkinson Building, Oxford Road, Manchester, M13 9PL.

Rebecca.mcphillips@manchester.ac.uk +44(0)161 275 5678

[1] Social Care and Society (SCS), Division of Population Health, Health Services Research and Primary Care, School of Health Sciences, Faculty of Biology, Medicine and Health, University of Manchester, Manchester, UK.

[2] Centre For Mental Health And Society, Bangor University, Wales.

[3] Chief Executive Officer, Pakistan Institute of Living and Learning, Karachi, Pakistan

Word count: 2241

For peer review only - http://bmjopen.bmj.com/site/about/guidelines.xhtml

ABSTRACT

Introduction: Over 800,000 people die due to suicide each year and suicide presents huge psychological, economic and social burdens for individuals, communities and countries as a whole. Low and middle income countries (LMICs) are disproportionately affected by suicide. The strongest risk factor for suicide is a previous suicide attempt, and other types of self-harm have been found to be robust predictors of suicidal behaviour. An approach that brings together multiple sectors including education, labour, business, law, politics and the media is crucial to tackling suicide and self-harm. The World Health Organization highlights that evaluations of the knowledge and attitudes that priority groups, not only healthcare staff, have of mental health and suicidal behaviour are key to suicide prevention strategies. The aim of this systematic review is to examine the knowledge, attitudes and experiences different stakeholders in LMICs have of self-harm and suicide.

Methods and analysis: Medline, Embase, PsycInfo, CINAHL, BNI, Social Sciences and Cochrane library will be searched. Reviewers working independently of each other will screen search results, select studies for inclusion, extract and check extracted data, and rate the quality of the studies using the STROBE and CASP checklists. In anticipation of heterogeneity, a narrative synthesis of quantitative studies will be provided and meta-ethnography will be used to synthesise qualitative studies.

Ethics and dissemination*:* Ethical approval is not required. A report will be provided for the funding body, and the systematic review will be submitted for publication in a high-impact, peer-reviewed, open access journal. Results will also be disseminated at conferences, seminars, congresses and symposia and to relevant stakeholders.

PROSPERO registration number: CRD42019135323

Strengths and limitations of this study

For peer review only - http://bmjopen.bmj.com/site/about/guidelines.xhtml

- A strength of this systematic review protocol is that it has been written according to the Preferred Reporting Items for Systematic review and Meta-Analysis (PRISMA-P) 2015 checklist.
- A strength of the review is that it will conform to the PRISMA statement and to the Cochrane systematic review literature guidelines when results are reported
- A strength of this review is that both quantitative and qualitative evidence will be assessed.
- A limitation of the review is the inclusion of peer reviewed studies only, however language restrictions will not be applied
- We anticipate that the quantitative studies included in the review will be heterogenous, therefore a limitation will be the lack of meta-analysis

Keywords

Self-harm; suicide; attitudes; knowledge; experience; quantitative; qualitative; low and middle income countries, LMICs

INTRODUCTION

The World Health Organization (WHO) estimate that over 800,000 people die due to suicide each year; 1 person every 40 seconds [1]. Suicide disproportionately affects low and middle income countries (LMICs), in 2014 the WHO reported that 75.5% of suicides globally occur in LMICs, and in South East Asia suicide is the leading cause of death in 15-29 year olds [1]. However, the under-reporting and misclassification of suicide as a cause of death in LMICs mean that suicide rates are likely higher than reported [1, 2]. Every suicide death is a tragedy for families, friends and communities and suicide presents huge psychological, economic and social burdens for individuals, communities and countries as a whole [1]. Reducing suicide is a key indicator for the United Nations sustainable development goal to ensure healthy lives and promote well-being at all ages globally [3]. However, much of the published literature on suicide relates to high income countries (HICs), and to effect change a better understanding of suicide within the cultural, political and socio-economic

For peer review only

context of LMICs is needed. Patient profiles, suicide rates, aetiology and methods differ between LMICs and HICs [4]. For example, research to date indicates that the ratio of women to men who die by suicide in LMICs is much lower than in HICs [5]. Furthermore, where marriage is considered to be a protective factor for women in HICs, it is less so for women in some LMICs, and self-immolation and the consumption of pesticides are far more common methods in LMICs than in HICs [6-9].

The strongest risk factor for suicide is a previous suicide attempt, and the WHO suggest that for each adult who dies from suicide there may be 20 others attempting suicide [1]. Suicide attempts and suicide are types of self-harm that are often differentiated from non-suicidal self-injury (NSSI) in terms of intent, frequency, methods, lethality and cognitions [10]. While the intent of suicidal behaviours is to kill oneself, the intent of NSSI is not. NSSI behaviours are more frequent than suicide and suicide attempts, with individuals employing more varying and less lethal methods and it is suggested that the cognitions related to NSSI concern temporary relief while those related to suicidal behaviour concern permanent relief [10-13]. Similarly to the literature on suicide, much of that concerning self-harm is focussed on HICs [14-16], where self-harm has been found to be a robust predictor of suicidal behaviour, with this link remaining after controlling for age, gender, and ethnicity [12, 17-18. A systematic review of the limited empirical research on self-harm in LMICs found that the prevalence of NSSI and suicide attempts in LMICs was comparable to HICs and that the most common methods of NSSI in LMICs were hitting, cutting, wound picking and biting and these findings were similar to evidence from HICs [16] Risk factors identified for self-harm in LMICs were often family related, for example family conflict, divorced parents and childhood abuse, and protective factors were high family functioning and understanding parents, which were attributed to greater reliance on family in LMICs compared to many Western HICs [16].

Suicide and self-harm in both LMICs and HICs are the result of complex interactions between genetic, psychological, biological, cultural, sociodemographic and social factors [1, 19-20]. Although the healthcare sector clearly has a vital role to play in tackling suicide and self-harm in LMICs, an approach that brings together multiple sectors including education, labour, business, law, politics and

For peer review only - http://bmjopen.bmj.com/site/about/guidelines.xhtml

the media is crucial [1, 21]. The knowledge, attitudes and experiences stakeholders from various sectors have of suicide and self-harm are likely to influence suicide and self-harm prevention and intervention strategies. A recent review by the WHO [21] highlights that evaluations of the knowledge and attitudes that priority groups, for example policy makers and community groups, not only healthcare staff, have of mental health and suicidal behaviour are key to the collection of high quality surveillance data and prevention strategies. Reviews to date have focused on the knowledge, attitudes and experiences that healthcare professionals have towards self-harm and suicide [22-25]. The aim of this systematic review is to examine various stakeholders' knowledge, attitudes and experiences of self-harm and suicide. Therefore, in addition to stakeholders from the healthcare sector, other stakeholders who will be included in this review are people who have experienced self-harm and/or have attempted suicide themselves, and their relatives, friends, and co-workers, and stakeholders from the social, healthcare, government, and criminal justice sectors. We are interested in exploring the range of publications on the broad spectrum of knowledge, attitudes and experiences that these various stakeholders may have concerning suicide and self-harm, including for example, knowledge stakeholders may have on prevalence and risk and protective factors for suicide and self-harm, stigmatising or empathetic attitudes towards those who self-harm, and experiences such as providing or receiving medical treatment for self-harm. This systematic review is being undertaken as part of the South Asia Self Harm Initiative (SASHI) project, which aims to help to find effective responses to self-harm and suicide in South Asia by building capability and capacity in research infrastructure and expertise in the region. Findings from this systematic review will be used to inform the development of a survey on knowledge, attitudes and well-being in South Asia. Thus, we are particularly interested in studies conducted in South Asia and countries with comparable healthcare systems or cultural backgrounds.

Research question

The SPICE (**S**etting, **P**erspective, phenomena of **I**nterest, **C**omparison, **E**valuation) framework was used to generate the research question that will be addressed by this systematic review [26]:

For peer review only

For peer review only - http://bmjopen.bmj.com/site/about/guidelines.xhtml

- What are stakeholders' knowledge, attitudes and experiences of self-harm and suicide in LMICs?

METHODS AND ANALYSIS

This protocol conforms to the Preferred Reporting Items for Systematic Reviews and Meta-Analyses Protocols (PRISMA-P) checklist (see Supplementary File 1) [27]. We will conform to the PRISMA statement and to the Cochrane systematic review literature guidelines when reporting the results [28-29]. This systematic review has been registered on PROSPERO [30].

Search strategy

A Betsi Cadwaladr University Health Board (BCUHB) librarian with expertise in systematic reviews has assisted the authors in the development of the search strategy (see Appendix 1). We will search Medline, Embase, PsycInfo, CINAHL, BNI, Social Sciences and Cochrane library. We will not apply any language restrictions to the search criteria. EndNote and Microsoft Word will be used to manage initial search results, screening and data throughout the review. We will update the searches prior to publication to ensure the latest papers are included. Reference lists from included studies and any identified systematic or literature reviews will also be searched by hand. Study authors will be contacted in instances when it has not been possible to retrieve full text articles and when clarification regarding inclusion criteria e.g. participant age, is required.

Study selection criteria

Inclusion criteria are empirical studies conducted in LMICs, as defined by the Organisation for Economic Co-operation and Development [31], irrespective of the study design, whose focus is on the knowledge, attitudes or experiences of stakeholders towards self-harm and/or suicide, where participants are aged 16 years and above. Studies that include stakeholders' knowledge, attitudes and experiences of suicide and self-harm related to those under 16 will be included. Stakeholders are people who have experienced self-harm and/or have attempted suicide themselves, relatives, friends,

For peer review only - http://bmjopen.bmj.com/site/about/guidelines.xhtml

co-workers, and healthcare workers of those who have self-harmed, attempted or completed suicide and people in the social, healthcare, government, and criminal justice sectors. Exclusion criteria are studies conducted in high income countries (HICs) and studies whose participants are not aged 16 years and above. Studies whose main focus is on the prevalence and/or predictors of self-harm and/or suicide, relationships between state and/or trait characteristics and self-harm and/or suicide, euthanasia, terrorism, or epidemiology will also be excluded. Systematic and literature reviews will be consulted for relevant references but will not be included in the review. Opinion pieces, editorials, book reviews, and conference and poster abstracts will not be included in the review.

The selection of studies for inclusion will adhere to the Cochrane guidelines and the process of selection of eligible studies will be illustrated via a PRISMA diagram [29]. Following deduplication of search results in EndNote, the following screening process will be undertaken in order to select studies for inclusion in the systematic review:

1) Titles and abstracts will be read by two reviewers independently, and relevance and fit with the inclusion criteria will be assessed. Those of no obvious relevance will be excluded and any disagreements will be resolved with a third reviewer (and the wider expert group if necessary).

2) Full text articles of remaining studies will be retrieved and read by two reviewers independently to assess their suitability for inclusion in the final review, disagreements will be resolved by discussion with a third reviewer (and the wider expert group if necessary). Both reviewers will populate a piloted pro-forma for each full text paper read (see Appendix 2).

Data extraction

For peer review only - http://bmjopen.bmj.com/site/about/guidelines.xhtml

Data will be extracted from selected studies by one reviewer, and a second reviewer will check for accuracy. Extracted data will be recorded on a piloted pro-forma (see Appendix 2), and will reflect the inclusion criteria and the designated aims of the review, derived from the article as a whole. Discrepancies will be resolved through discussion (with the wider expert group if necessary). Additional data will be requested from study authors when necessary. Data extraction of qualitative studies (and for qualitative components in studies with mixed methods) will adhere to the same methods and will be reviewed independently.

For peer review only

Outcomes

Outcomes of interest include:

- The identification of relevant information on stakeholders' knowledge, attitudes and experiences of self-harm and suicide, particularly in South Asia and in countries with comparable healthcare systems and cultural backgrounds
- The quantitative methods and measures that have been used to investigate stakeholders' attitudes towards and knowledge about self-harm and suicide and their psychometric properties
- The qualitative methods that have been used to investigate stakeholders' attitudes towards, knowledge about, and experiences of self-harm and suicide.

The identified outcomes will inform the development of a survey on knowledge, attitudes and well-being in South Asia as part of the SASHI project.

Quality assessment

All eligible studies will be subject to quality appraisal. The quality of included quantitative studies will be appraised using the STROBE checklist [32]. The STROBE Statement consists of a checklist of 22 items, which relate to the title, abstract, introduction, methods, results and discussion sections of articles. Eighteen items are common to cohort studies, case-control studies and cross-sectional studies and four are specific to each of the three study designs. The quality of included qualitative studies will

For peer review only - http://bmjopen.bmj.com/site/about/guidelines.xhtml

be appraised using the CASP checklist [33]. The 10-item CASP tool was considered to be the most suitable tool to consider the quality parameters of qualitative work, and is a well-validated and accepted tool [28]. Both the STROBE and CASP checklists will be applied independently by two reviewers and any disagreements will be resolved with a third reviewer (and the wider expert group if necessary).

Studies will not be excluded on the basis of poor quality alone, rather all studies that meet the inclusion criteria will be included in the review. This low threshold for inclusion will be applied so that the review can benefit from researcher insight and theoretical as well as empirical contributions. The relative quality of included studies will be critically considered and discussed in the review.

Descriptive analysis and data synthesis

We anticipate that the quantitative studies included in the review will be heterogenous and this will prevent meta-analysis. We will provide a narrative synthesis of quantitative studies, structured around population characteristics and the geographical region of studies. We will provide summaries of the quantitative methods and measures used to investigate stakeholders' attitudes towards and knowledge about self-harm and suicide and their psychometric properties.

Meta-ethnography will be used to synthesise qualitative studies [34]. Initially reciprocal translation will be performed by comparing the concepts presented in different studies. A chronological approach will be taken to reciprocal translation; studies will be arranged chronologically, concepts from papers one and two will be compared, and the synthesis of papers one and two will then be compared with paper three, and so forth, as is described elsewhere [35]. When contradictions between studies are identified, we will perform refutational synthesis by exploring and explaining these. A 'lines-of-argument' synthesis, that links and explains concepts presented by different studies, will be conducted so that an interpretation of all included studies can be presented.

For peer review only - http://bmjopen.bmj.com/site/about/guidelines.xhtml

Two reviewers will lead data synthesis. Emergent analysis, and any discrepancies, will be discussed with other members of the review team. Microsoft Office software will be used to facilitate data synthesis.

Patient and public involvement

No patients or members of the public were involved in the design of this study.

Amendments

An amendment has been made to the initial registration of this systematic review in PROSPERO, which details that only studies from LMICs will be included in this review, and studies from HICs will be excluded from this review. Any further amendments to this protocol will be documented in the full review.

ETHICS AND DISSEMINATION

Ethics approval is not required as this is a protocol for the systematic review of previously published data. In addition to a report to the funding body, we intend to submit the systematic review for publication in a high-impact, peer-reviewed journal. We will select an open access journal to ensure free access to undergraduate and graduate students, researchers, academics and research groups. Results will also be disseminated at conferences seminars, congresses and symposia and to relevant stakeholders.

Acknowledgements: The authors would like to thank Mrs Nia Morris (NM) from BCUHB library for her contribution to the development of the search strategy.

Contributions: CR, RM, PH, AK, MK conceived the research idea. SN led the development of the search strategy, with input from NM, RM, AK, PH. RM, SN, MK, AK, PH, CR conceived the manuscript. RM lead the drafting of the manuscript with contributions from SN, AE, SB, MK, AK, PH, NC, CR. All authors approved the final manuscript.

For peer review only - http://bmjopen.bmj.com/site/about/guidelines.xhtml

Funding: This manuscript was funded by UK Research and Innovation Global Challenge Research Fund (ref: MR/P028144/1). The finder did not have any role in developing this protocol.

Competing interests: None declared

References

[1] World Health Organization. Preventing suicide: a global imperative. World Health Organization, 2014. Available: https://apps.who.int/iris/bitstream/handle/10665/131056/9789241564779_eng.pdf;jsessionid=F6255CAAD6496DA078DE41FAB0C5CCBF?sequence=1 . [Accessed 04 May 2020].

[2] Jordans MJD, Kaufman A, Brenman NF, *et al*. Suicide in South Asia: a scoping review. *BMC Psychiatry* 2014;**14**:358.doi:10.1186/s12888-014-0358-9 pmid: https://www.ncbi.nlm.nih.gov/pubmed/25539951

[3] United Nations. Department of economic and social affairs sustainable development: Ensure healthy lives and promote well-being for all at all ages. United Nations, 2015. Available: https://sdgs.un.org/goals/goal3 [Accessed 20 December 2020]

[4] Ward E, McCartney T, Arscott-Mills S, *et al*. The Jamaica injury surveillance system: a profile of the intentional and unintentional injuries in Jamaican hospitals. *West Indian Med J* 2010;**59**:7-13. pmid: https://www.ncbi.nlm.nih.gov/pubmed/20931906

[5] World Health Organization. Suicide in the World: global health estimates. World Health Organization, 2019. Available: https://www.who.int/publications/i/item/suicide-in-the-world [Accessed 20 December 2020]

For peer review only - http://bmjopen.bmj.com/site/about/guidelines.xhtml

[6] Aggarwal S. Suicide in India. *Br Med Bull* 2015;**114**:127-134.doi:10.1093/bmb/ldv018 pmid: https://www.ncbi.nlm.nih.gov/pubmed/25958380

[7] Gururaj G, Isaac MK, Subbakrishna DK, *et al*. Risk factors for completed suicides: a case-control study from Bangalore, India. *Inj Control Saf Promot* 2004;**3**:183-191.doi:10.1080/156609704/233/289706 pmid: https://www.ncbi.nlm.nih.gov/pubmed/15764105

[8] Laloe V. Patterns of deliberate self-burning in various parts of the world: a review. *Burns* 2004;**30**:207-215.doi:10.1016/j.burns.2003.10.018 pmid: https://www.ncbi.nlm.nih.gov/pubmed/15082345

[9] Vijayakumar L. Suicide in women. *Indian J Psychiatry* 2015;**57**:S233-S238.doi:10.4103/0019-5545.161484 pmid: https://www.ncbi.nlm.nih.gov/pubmed/26330640

[10] Jacobson CM, Gould M. The epidemiology and phenomenology of non-suicidal self-injurious behavior among adolescents: a critical review of the literature. *Arch Suicide Res* 2007;**11**:12-47doi:10.1080/13811110701247602 pmid: https://www.ncbi.nlm.nih.gov/pubmed/17453692

[11] Muehlenkamp JJ. Self-injurious behavior as a separate clinical syndrome. *Am J Orthopsychiatry* 2005;**75**:324-333.doi:10.1037/0002-9432.75.2.324 pmid: https://www.ncbi.nlm.nih.gov/pubmed/15839768

[12] Nock MK. Self-injury. *Annu Rev Clin Psychol* 2010;**6**:339-363 doi:10.1146/annurev.clinpsy.121208.131258. pmid: https://www.ncbi.nlm.nih.gov/pubmed/20192787

For peer review only

[13] Walsh BW. *Treating self-injury: A practical guide* . New York, USA: Guilford Press 2005

[14] Gholamrezaei M, Stefano JD, Heath NL. Nonsuicidal self-injury across cultures and ethnic and racial minorities: a review. *Int J Psychol* 2017;**52**:316-326. doi:10.1002/ijop.12230 pmid: https://www.ncbi.nlm.nih.gov/pubmed/26644040

[15] Aggerwal S, Berk M. Nonsuicidal self-injury in Indian adolescents: nonexistent or unacknowledged? *Int J Soc Psychiatry* 2015;**61**:516-517doi:10.1177/0020764015579739 pmid: https://www.ncbi.nlm.nih.gov/pubmed/26209701

[16] Aggarwal S, Patton G, Reavley N. Youth self-harm in low- and middle- income countries: systematic review of the risk and protective factors. *Int J Soc Psychiatry* 2017;**63**:359-375doi: 10.1177/0020764017700175 pmid: https://www.ncbi.nlm.nih.gov/pubmed/ 28351292

[17] Hamza CA, Stewart SL, Willoughby T. Examining the link between nonsuicidal self-injury and suicidal behaviour: a review of the literature and integrated model. *Clin Psychol Rev* 2012;**6** 482-95.doi:10.1016/j.cpr.2012.05.003. pmid: https://www.ncbi.nlm.nih.gov/pubmed/22717336

[18] Owens D, Horrocks J, House A. Fatal and non-fatal repetition of self-harm: systematic review. *Br J Psychiatry* 2002;**181**:193-99. doi: 10.1192/bjp.181.3.193 pmid: https://www.ncbi.nlm.nih.gov/pubmed/12204922

[19] Hawton K, Sanders KEA, O'Connor RC. Self-harm and suicide in adolescents. *Lancet* 2012;**379**:2373-82. doi: 10.1016/S0140-6736(12)60322-5 pmid: https://www.ncbi.nlm.nih.gov/pubmed/22726518

For peer review only - http://bmjopen.bmj.com/site/about/guidelines.xhtml

[20] Yip PSF, Caine E, Yousef S, *et al*. Means restriction for suicide prevention. *Lancet* 2012;379:2393-2399. doi: 10.1016/S0140-6736(12)60521-2

[21] World Health Organization. National suicide prevention strategies: progress, examples and indicators. World Health Organization, 2018. Available: https://apps.who.int/iris/bitstream/handle/10665/279765/9789241515016-eng.pdf?ua=1 . [accessed 04 May 2020].

[22] Karman P, Kool N, Poslawsky IE, *et al.* Nurses' attitudes towards self-harm: a literature review. *J Psychiatr Ment Health Nurs* 2015;**22**:65-75. doi: 10.1111/jpm.12171 pmid: https://www.ncbi.nlm.nih.gov/pubmed/25490929

[23] O'Connor S, Glover L. Hospital staff experiences of their relationships with adults who self-harm: a meta-synthesis. *Psychol Psychother* 2017;**90**:480-501. doi: 10.1111/papt.12113. pmid: https://www.ncbi.nlm.nih.gov/pubmed/28035740

[24] Rees N, Rapport F, Thomas G, *et al*. Perceptions of paramedic and emergency care workers of those who self-harm: a systematic review of the quantitative literature. *J Psychosom Res* 2014:**77**;449-456. doi: 10.1016/j.jpsychores.2014.09.006. pmid: https://www.ncbi.nlm.nih.gov/pubmed/25263398

[25] Saunders KEA, Hawton K, Fortune S, *et al*. Attitudes and knowledge of clinical staff regarding people who self-harm: a systematic review. *J Affect Disord* 2012;**139**:205-216. doi: 10.1016/j.jad.2011.08.024 pmid: https://www.ncbi.nlm.nih.gov/pubmed/21925740

[26] Booth A. Clear and present questions: formulating questions for evidence based practice. *Library Hi Tech* 2006;**24**:355-368. doi: 10.1108/07378830610692127

For peer review only - http://bmjopen.bmj.com/site/about/guidelines.xhtml

[27] Shamseer L, Moher D, Clarke M, *et al*. Preferred reporting items for systematic review and meta-analysis protocols (PRISMA-P) 2015: elaboration and explanation. *BMJ* 2015;**350**:g7647. doi: 10.1136/bmj.g7647 pmid: https://www.ncbi.nlm.nih.gov/pubmed/25555855

[28] Higgins JPT, Green S (editors). Cochrane handbook for systematic reviews of interventions Version 6. The Cochrane Collaboration, 2019. Available: http://handbook.cochrane.org. [Accessed 04 May 2020].

[29] Liberati A, Altman DG, Tetzlaff J, *et al*. The PRISAM statement for reporting systematic reviews and meta-analyses of studies that evaluate healthcare interventions: explanation and elaboration. *BMJ* 2009;**339**:b2700. doi: 10.1136/bmj.b2700 pmid: https://www.ncbi.nlm.nih.gov/pubmed/19622552

[30] McPhillips R, Nafees S, Krishna M, *et al*. Knowledge, attitudes, and experiences of self-harm and suicide in low and middle income countries: a systematic review. PROSPERO 2019 CRD42019135323. Available: https://www.crd.york.ac.uk/prospero/display_record.php?ID=CRD42019135323 [Accessed 04 May 2020]

[31] Organisation for Economic Co-operation and Development. DAC List of ODA recipients for reporting on aid in 2018 and 2019. Organisation for Economic Co-operation and Development, 2019. Available: http://www.oecd.org/dac/financing-sustainable-development/development-finance-standards/daclist.htm [Accessed 04 May 2020]

[32] von Elm E, Altman DG, Egger M, *et al.* STROBE Initiative. The strengthening the reporting in observational studies in epidemiology (STROBE) statement guidelines for reporting observational studies. Lancet 2007;**370**:1453-57 doi: 0.1016/S0140-6736(07)61602-X pmid: https://www.ncbi.nlm.nih.gov/pubmed/18064739

For peer review only - http://bmjopen.bmj.com/site/about/guidelines.xhtml

[33] Critical Appraisal Skills Programme. CASP qualitative checklist. Critical Appraisal Skills Programme , 2018. Available: https://casp-uk.net/wp-content/uploads/2018/01/CASP-Qualitative-Checklist-2018.pdf
[Accessed: 04 May 2020]

[34Noblit GW, Hare RD. Meta-ethnography: synthesising qualitative studies. Sage Publications.

[35] Atkins S, Lewin S, Smith H *et al*. Conducting a meta-ethnography of qualitative literature: lessons learnt. *BMC Med Res Methodol* 2008;**8**:21. doi: 10.1186/1471-2288-8-21 pmid: https://www.ncbi.nlm.nih.gov/pubmed/ 18416812

For peer review only

For peer review only - http://bmjopen.bmj.com/site/about/guidelines.xhtml

For peer review only

For peer review only - http://bmjopen.bmj.com/site/about/guidelines.xhtml

Appendix 1: Knowledge, attitudes, and experiences of self-harm and suicide in low and middle income countries: Medline search strategy

Self-harm and suicide terms

1 Suicide/
2 Suicide.mp
3 Suicide, Attempted/
4 Suicide, Attempted.mp
5 Self-Injurious Behavior/
6 Self-Injurious Behavior.mp
7 Self-Mutilation/
8 Self-Mutilation.mp
9 Suicidal Ideation/
10 Suicidal Ideation.mp
11 Attempted Suicide/
12 Attempted Suicide.mp
13 Drug Overdose/
14 Drug Overdose.mp
15 ((Self adj2 cut$) or (para adj suicid$) or (attempt$ adj suicide$) or (suicid$ adj behavio$)).mp.
16 (auto?mutilat$ or cutt$ or over?dos$ or self?destruct$ or self?harm$ or self?immolat$ or self?inflict$ or self?injur$ or self?mutilat$ or self?poison$ or suicide$ or self?burn$).mp.
17 Or/1-16

Stakeholder terms

18 exp Health Personnel/
19 Health Personnel.mp
20 exp PHYSICIANS/
21 PHYSICIANS.mp
22 exp Personnel, Hospital/
23 Personnel, Hospital.mp
24 Social Workers/
25 Social Workers.mp
26 ((doctor$ or physician$ or hosp$ or medic$ or health?care or social or wel?fare) adj (profession$ or staff$ or officer$ or person$ or worker$)).mp
27 Family/
28 Famil$.mp
29 Friends/
30 Friend$.mp
31 Caregivers/
32 Caregiver$.mp
33 Criminals/
34 Criminal$.mp
35 Prisoners/
36 Prison$.mp
37 Social Justice/
38 Soci$ adj Just$.mp
39 (partner$ or parent$ or grandparent$ or children or sibling$ or famil$ or friend$ or relative$).mp.
40 (communit$ or societ$ or government$).mp.
41 (criminal$ or offender$ or prisoner$ or in?mate$).mp.
42 (justice adj system$).mp.

For peer review only

43 or/18-42

Knowledge and attitude terms

44 Attitude/
45 Attitude of Health Personnel/
46 Attitude to Death/
47 Knowledge/
48 Health Knowledge, Attitudes, Practice/
49 Awareness/
50 Education/
51 Health Education/
52 ((train$ or trainer$ or experienc$ or perspectiv$ or knowledg$ or attitud$ or awaren$ or educat$) adj health).mp.
53 social behavior/ or help-seeking behavior/ or self-control/ or shyness/ or social adjustment/ or social isolation/ or social marginalization/ or social skills/ or social stigma/ or social exclusion/ or social inclusion
54 Prejudice/
55 Taboo/
56 exp Shame/
57 (tabo$ or sham$ or stigma$ or prejudice$ or help?seek$ behavior$ or self?control$ or shy$ or (social adj adjust$ or behavio$ or isolate$ or margin$ or Stigm$ or skil$)).mp
58 or/44-57

LMIC terms

59 Developing Countries/
60 "low and middle income countr$".ab,ti.
61 LMIC.mp.
62 india/ or sikkim/ or pakistan/
63 exp Asia/
64 or/59-63

Self-harm and suicide, stakeholders, knowledge and attitude and LMIC combined (with limits)

65 17 and 43 and 58 and 64
66 limit 65 to humans
67 limit 66 (adolescent <13 to 17 years> or adult <18 to 64 years> or aged <65+ years>) (aged or "aged, 80 and over" or "frail elderly" or "middle aged" or "young adult" or adults or "teenager" or adolescent).mp

For peer review only

Appendix 2: Knowledge, attitudes, and experiences of self-harm and suicide in low and middle income countries systematic review: data extraction form

Notes for reviewers

Record any missing information as unclear or not described to ensure it is clear that the information was not found in the paper, not that you forgot to extract it

**General information**		
Date form completed		
Reviewer extracting data		
Study title		
Study authors		
Journal		
Year of publication		
Study author contact details		
Notes		
**Study eligibility for inclusion in review**		
Main focus on stakeholders' knowledge, attitudes and experiences of self-harm and/or suicide (excluding euthanasia or terrorism)	*Yes/No*	*Location in text (pg. #)*
Study population aged 16 and above (or can data from those aged 16 and above only can be extracted?)	*Yes/No*	*Location in text (pg. #)*
Low-middle income country	*Yes/No*	*Location in text (pg. #)*
Include or exclude	*Include/Exclude*	
Reason for exclusion	*Reasons for exclusion*<br>*1) Knowledge, attitudes and experience of self-harm/suicide not main concern of study (including terrorism and euthanasia) include main phenomenon being studied in notes – to be reviewed after 25 studies*<br>*1a) Completely irrelevant topic e.g. paper on depression, no mention of self-harm/suicide 1b) Focus on prevalence of suicide/self-harm*<br>*1c) Focus on risk factors of suicide/self-harm*<br>*1d) Focus on intervention only*<br>*1e) Mention of self-harm/suicide however topic not relevant to attitudes, knowledge and experiences of self-harm/suicide*<br>*2) Research not conducted in LMICs*<br>*3) Research population not 16 and over*<br>*4) Literature review*<br>*5) Commentary, book review, editorial*	
Notes		
**Characteristics of included studies: Participants**		

For peer review only

	Description as stated in paper	*Location in text (pg. #)*
Study location (Country and state/city/area) e.g. India, Bangalore		
Study setting e.g. hospital, community		
Study population e.g. nurses, community members		
Informed consent obtained	*Yes, No, Unclear*	
Total number of participants		
Exclusions and withdrawals		
Participant demographics e.g.<br>Age<br>Sex<br>Race/Ethnicity<br>Religious beliefs<br>Mental illness diagnosis<br>Physical illness diagnosis<br>Other demographics		
Notes		
**Characteristics of included studies: Methods**		
	Description as stated in paper	*Location in text (pg. #)*
Aim of study/Research question(s) (implicit or explicit in text?)		
Study methodology (or methodologies)		
Quantitative measures used e.g. Acceptability of Suicide Scale and information on whether measure is validated (if applicable)		
Quantitative analysis methods and procedure		
Qualitative methods used e.g. focus group, one-to-one interviews, vignettes (if applicable)		
Theoretical/epistemological perspectives underpinning qualitative research (explicit or reviewer's interpretation)		
Qualitative data analysis methods and procedure		
Start date and end date		
Notes		
**Characteristics of included studies: Results**		
	Description as stated in paper	*Location in text (pg. #)*
Qualitative results – direct quotes from participants (first order)		

For peer review only

Qualitative results – study author's interpretations of data (second order)		
Quantitative results		
Indicators of acceptability to users (if applicable)		
Suggested mechanisms of intervention action (if applicable)		
**Characteristics of included studies: Other information**		
	Description as stated in paper	*Location in text (pg. #)*
Key conclusions of authors		
References to other relevant studies		
Correspondence required by reviewers for further information (who, when, what requested)		
Notes		

For peer review only

Supplementary file 1: PRISMA-P (Preferred Reporting Items for Systematic review and Meta-Analysis Protocols) 2015 checklist: recommended items to address in a systematic review protocol*

Section and topic	Item No	Checklist item	Complete
**ADMINISTRATIVE INFORMATION**			
Title:			
Identification	1a	Identify the report as a protocol of a systematic review	X
Update	1b	If the protocol is for an update of a previous systematic review, identify as such	N/A
Registration	2	If registered, provide the name of the registry (such as PROSPERO) and registration number	X
Authors:			
Contact	3a	Provide name, institutional affiliation, e-mail address of all protocol authors; provide physical mailing address of corresponding author	X
Contributions	3b	Describe contributions of protocol authors and identify the guarantor of the review	X
Amendments	4	If the protocol represents an amendment of a previously completed or published protocol, identify as such and list changes; otherwise, state plan for documenting important protocol amendments	X
Support:			
Sources	5a	Indicate sources of financial or other support for the review	X
Sponsor	5b	Provide name for the review funder and/or sponsor	X
Role of sponsor or funder	5c	Describe roles of funder(s), sponsor(s), and/or institution(s), if any, in developing the protocol	X
**INTRODUCTION**			
Rationale	6	Describe the rationale for the review in the context of what is already known	X
Objectives	7	Provide an explicit statement of the question(s) the review will address with reference to participants, interventions, comparators, and outcomes (PICO)	X
**METHODS**			
Eligibility criteria	8	Specify the study characteristics (such as PICO, study design, setting, time frame) and report characteristics (such as years considered, language, publication	X

For peer review only

		status) to be used as criteria for eligibility for the review	
Information sources	9	Describe all intended information sources (such as electronic databases, contact with study authors, trial registers or other grey literature sources) with planned dates of coverage	X
Search strategy	10	Present draft of search strategy to be used for at least one electronic database, including planned limits, such that it could be repeated	X
Study records:			
Data management	11a	Describe the mechanism(s) that will be used to manage records and data throughout the review	X
Selection process	11b	State the process that will be used for selecting studies (such as two independent reviewers) through each phase of the review (that is, screening, eligibility and inclusion in meta-analysis)	X
Data collection process	11c	Describe planned method of extracting data from reports (such as piloting forms, done independently, in duplicate), any processes for obtaining and confirming data from investigator	X
Data items	12	List and define all variables for which data will be sought (such as PICO items, funding sources), any pre-planned data assumptions and simplifications	X
Outcomes and prioritization	13	List and define all outcomes for which data will be sought, including prioritization of main and additional outcomes, with rationale	X
Risk of bias in individual studies	14	Describe anticipated methods for assessing risk of bias of individual studies, including whether this will be done at the outcome or study level, or both; state how this information will be used in data synthesis	X
Data synthesis	15a	Describe criteria under which study data will be quantitatively synthesised	X
	15b	If data are appropriate for quantitative synthesis, describe planned summary measures, methods of handling data and methods of combining data from studies, including any planned exploration of consistency (such as I^2, Kendall's τ)	N/A
	15c	Describe any proposed additional analyses (such as sensitivity or subgroup analyses, meta-regression)	X
	15d	If quantitative synthesis is not appropriate, describe the type of summary planned	X
Meta-bias(es)	16	Specify any planned assessment of meta-bias(es) (such as publication bias across studies, selective reporting within studies)	N/A
Confidence in cumulative evidence	17	Describe how the strength of the body of evidence will be assessed (such as GRADE)	N/A

For peer review only

*** It is strongly recommended that this checklist be read in conjunction with the PRISMA-P Explanation and Elaboration (cite when available) for important clarification on the items. Amendments to a review protocol should be tracked and dated. The copyright for PRISMA-P (including checklist) is held by the PRISMA-P Group and is distributed under a Creative Commons Attribution Licence 4.0.**

From: Shamseer L, Moher D, Clarke M, Ghersi D, Liberati A, Petticrew M, Shekelle P, Stewart L, PRISMA-P Group. Preferred reporting items for systematic review and meta-analysis protocols (PRISMA-P) 2015: elaboration and explanation. BMJ. 2015 Jan 2;349(jan02 1):g7647.

For peer review only

BMJ Open

Knowledge, attitudes, and experiences of self-harm and suicide in low and middle income countries: protocol for a systematic review

Journal:	*BMJ Open*
Manuscript ID	bmjopen-2020-041645.R2
Article Type:	Protocol
Date Submitted by the Author:	10-Mar-2021
Complete List of Authors:	McPhillips, Rebecca; The University of Manchester, Social Care and Society<br>Nafees, Sadia; Bangor University, North Wales Centre for Primary Care Research<br>Elahi, Anam ; The University of Manchester, Social Care and Society<br>Batool, Saqba; The University of Manchester, Social Care and Society<br>Krishna, Murali; Bangor University<br>Krayer, Anne; Bangor University<br>Huxley, Peter; Bangor University<br>Chaudhry, Nasim ; Pakistan Institute of Living and Learning<br>Robinson, Catherine; The University of Manchester
**Primary Subject Heading**:	Global health
Secondary Subject Heading:	Mental health
Keywords:	Suicide & self-harm < PSYCHIATRY, PUBLIC HEALTH, MENTAL HEALTH

SCHOLARONE™
Manuscripts

I, the Submitting Author has the right to grant and does grant on behalf of all authors of the Work (as defined in the below author licence), an exclusive licence and/or a non-exclusive licence for contributions from authors who are: i) UK Crown employees; ii) where BMJ has agreed a CC-BY licence shall apply, and/or iii) in accordance with the terms applicable for US Federal Government officers or employees acting as part of their official duties; on a worldwide, perpetual, irrevocable, royalty-free basis to BMJ Publishing Group Ltd ("BMJ") its licensees and where the relevant Journal is co-owned by BMJ to the co-owners of the Journal, to publish the Work in this journal and any other BMJ products and to exploit all rights, as set out in our licence.

The Submitting Author accepts and understands that any supply made under these terms is made by BMJ to the Submitting Author unless you are acting as an employee on behalf of your employer or a postgraduate student of an affiliated institution which is paying any applicable article publishing charge ("APC") for Open Access articles. Where the Submitting Author wishes to make the Work available on an Open Access basis (and intends to pay the relevant APC), the terms of reuse of such Open Access shall be governed by a Creative Commons licence – details of these licences and which Creative Commons licence will apply to this Work are set out in our licence referred to above.

Other than as permitted in any relevant BMJ Author's Self Archiving Policies, I confirm this Work has not been accepted for publication elsewhere, is not being considered for publication elsewhere and does not duplicate material already published. I confirm all authors consent to publication of this Work and authorise the granting of this licence.

For peer review only

Knowledge, attitudes, and experiences of self-harm and suicide in low and middle income countries: protocol for a systematic review

Rebecca McPhillips*[1]

Sadia Nafees[2]

Anam Elahi[1]

Saqba Batool[1]

Murali Krishna[2]

Anne Krayer[2]

Peter Huxley[2]

Nasim Chaudhry[3],

Catherine Robinson[1]

*Room C4.5, Ellen Wilkinson Building, Oxford Road, Manchester, M13 9PL.

Rebecca.mcphillips@manchester.ac.uk +44(0)161 275 5678

[1] Social Care and Society (SCS), Division of Population Health, Health Services Research and Primary Care, School of Health Sciences, Faculty of Biology, Medicine and Health, University of Manchester, Manchester, UK.

[2] Centre For Mental Health And Society, Bangor University, Wales.

[3] Chief Executive Officer, Pakistan Institute of Living and Learning, Karachi, Pakistan

Word count: 2241

For peer review only

ABSTRACT

Introduction: Over 800,000 people die due to suicide each year and suicide presents huge psychological, economic and social burdens for individuals, communities and countries as a whole. Low and middle income countries (LMICs) are disproportionately affected by suicide. The strongest risk factor for suicide is a previous suicide attempt, and other types of self-harm have been found to be robust predictors of suicidal behaviour. An approach that brings together multiple sectors including education, labour, business, law, politics and the media is crucial to tackling suicide and self-harm. The World Health Organization highlights that evaluations of the knowledge and attitudes that priority groups, not only healthcare staff, have of mental health and suicidal behaviour are key to suicide prevention strategies. The aim of this systematic review is to examine the knowledge, attitudes and experiences different stakeholders in LMICs have of self-harm and suicide.

Methods and analysis: Medline, Embase, PsycInfo, CINAHL, BNI, Social Sciences and Cochrane library will be searched. Reviewers working independently of each other will screen search results, select studies for inclusion, extract and check extracted data, and rate the quality of the studies using the STROBE and CASP checklists. In anticipation of heterogeneity, a narrative synthesis of quantitative studies will be provided and meta-ethnography will be used to synthesise qualitative studies.

Ethics and dissemination*:* Ethical approval is not required. A report will be provided for the funding body, and the systematic review will be submitted for publication in a high-impact, peer-reviewed, open access journal. Results will also be disseminated at conferences, seminars, congresses and symposia and to relevant stakeholders.

PROSPERO registration number: CRD42019135323

Strengths and limitations of this study

For peer review only - http://bmjopen.bmj.com/site/about/guidelines.xhtml

- A strength of this systematic review protocol is that it has been written according to the Preferred Reporting Items for Systematic review and Meta-Analysis (PRISMA-P) 2015 checklist.
- A strength of the review is that it will conform to the PRISMA statement and to the Cochrane systematic review literature guidelines when results are reported
- A strength of this review is that both quantitative and qualitative evidence will be assessed.
- A limitation of the review is the inclusion of peer reviewed studies only, however language restrictions will not be applied
- As it is likely that the quantitative studies included in the review will be heterogenous, therefore a limitation will be the lack of meta-analysis

Keywords

Self-harm; suicide; attitudes; knowledge; experience; quantitative; qualitative; low and middle income countries, LMICs

INTRODUCTION

The World Health Organization (WHO) estimate that over 800,000 people die due to suicide each year; 1 person every 40 seconds [1]. Suicide disproportionately affects low and middle income countries (LMICs), in 2014 the WHO reported that 75.5% of suicides globally occur in LMICs, and in South East Asia suicide is the leading cause of death in 15-29 year olds [1]. However, the under-reporting and misclassification of suicide as a cause of death in LMICs mean that suicide rates are likely higher than reported [1, 2]. Every suicide death is a tragedy for families, friends and communities and suicide presents huge psychological, economic and social burdens for individuals, communities and countries as a whole [1]. Reducing suicide is a key indicator for the United Nations sustainable development goal to ensure healthy lives and promote well-being at all ages globally [3]. However, much of the published literature on suicide relates to high income countries (HICs), and to effect change a better understanding of suicide within the cultural, political and socio-economic

For peer review only

context of LMICs is needed. Patient profiles, suicide rates, aetiology and methods differ between LMICs and HICs [4]. For example, research to date indicates that the ratio of women to men who die by suicide in LMICs is much lower than in HICs [5]. Furthermore, while marriage is considered to be a protective factor for women in HICs, it is less so for women in some LMICs, and self-immolation and the consumption of pesticides are far more common methods in LMICs than in HICs [6-9].

The strongest risk factor for suicide is a previous suicide attempt, and the WHO suggest that for each adult who dies from suicide there may be 20 others attempting suicide [1]. Harm arising from suicidal behaviour, suicide attempts and suicide are types of self-harm that are often differentiated from non-suicidal self-injury (NSSI) in terms of intent, frequency, methods, lethality and cognitions [10]. The motivation for suicidal behaviours is often to remove suffering ande the intent of suicidal behaviours is to end one's life, whereas the intent of NSSI is not. NSSI behaviours are more frequent than suicide and suicide attempts, with individuals employing more varying and less lethal methods, and it is suggested that the cognitions related to NSSI concern temporary relief while those related to suicidal behaviour concern permanent relief [10-13]. Similarly to the literature on suicide, much of that concerning NSSI is focussed on HICs [14-16], where NSSI has been found to be a robust predictor of suicidal behaviour, with this link remaining after controlling for age, gender, and ethnicity [12, 17-18]. A systematic review of the limited empirical research on self-harm, including suicidal self-harm and NSSI, in LMICs found that the prevalence of NSSI and suicide attempts in LMICs was comparable to HICs, that the most common methods of NSSI in LMICs were hitting, cutting, wound picking and biting and these findings were similar to evidence from HICs [16] Risk factors identified for suicidal self-harm and NSSI in LMICs were often family related, for example family conflict, divorced parents and childhood abuse, and protective factors were high family functioning and understanding parents, which were attributed to greater reliance on family in LMICs compared to many Western HICs [16].

Suicide and self-harm in both LMICs and HICs are the result of complex interactions between genetic, psychological, biological, cultural, sociodemographic and social factors [1, 19-20]. Although

For peer review only - http://bmjopen.bmj.com/site/about/guidelines.xhtml

the healthcare sector clearly has a vital role to play in tackling suicide and self-harm in LMICs, an approach that brings together multiple sectors including education, labour, business, law, politics and the media is crucial [1, 21]. The knowledge, attitudes and experiences stakeholders from various sectors have of suicide and self-harm are likely to influence suicide and self-harm prevention and intervention strategies. A recent review by the WHO [21] highlights that evaluations of the knowledge and attitudes that priority groups, for example policy makers and community groups, not only healthcare staff, have of mental health and suicidal behaviour are key to the collection of high quality surveillance data and prevention strategies. Reviews to date have focused on the knowledge, attitudes and experiences that healthcare professionals have towards self-harm and suicide [22-25]. The aim of this systematic review is to examine various stakeholders' knowledge, attitudes and experiences of self-harm and suicide. Therefore, in addition to stakeholders from the healthcare sector, other stakeholders who will be included in this review are people who have experienced self-harm and/or have attempted suicide themselves, and their relatives, friends, and co-workers, and stakeholders from the social, healthcare, government, and criminal justice sectors. We are interested in exploring the range of publications on the broad spectrum of knowledge, attitudes and experiences that these various stakeholders may have concerning suicide and self-harm, including for example, knowledge stakeholders may have on prevalence and risk and protective factors for suicide and self-harm, stigmatising or empathetic attitudes towards those who self-harm, and experiences such as providing or receiving medical treatment for self-harm. This systematic review is being undertaken as part of the South Asia Self Harm Initiative (SASHI) project, which aims to help to find effective responses to self-harm and suicide in South Asia by building capability and capacity in research infrastructure and expertise in the region. Findings from this systematic review will be used to inform the development of a survey on knowledge, attitudes and well-being in South Asia. Thus, we are particularly interested in studies conducted in South Asia and countries with comparable healthcare systems or cultural backgrounds.

Research question

For peer review only - http://bmjopen.bmj.com/site/about/guidelines.xhtml

The SPICE (**S**etting, **P**erspective, phenomena of **I**nterest, **C**omparison, **E**valuation) framework was used to generate the research question that will be addressed by this systematic review [26]:

- What are stakeholders' knowledge, attitudes and experiences of self-harm and suicide in LMICs?

METHODS AND ANALYSIS

This protocol conforms to the Preferred Reporting Items for Systematic Reviews and Meta-Analyses Protocols (PRISMA-P) checklist (see Supplementary File 1) [27]. We will conform to the PRISMA statement and to the Cochrane systematic review literature guidelines when reporting the results [28-29]. This systematic review has been registered on PROSPERO [30].

Search strategy

A Betsi Cadwaladr University Health Board (BCUHB) librarian with expertise in systematic reviews has assisted the authors in the development of the search strategy (see Appendix 1). We will search Medline, Embase, PsycInfo, CINAHL, BNI, Social Sciences and Cochrane library. We will not apply any language restrictions to the search criteria. EndNote and Microsoft Word will be used to manage initial search results, screening and data throughout the review. We will update the searches prior to publication to ensure the latest papers are included. Reference lists from included studies and any identified systematic or literature reviews will also be searched by hand. Study authors will be contacted in instances when it has not been possible to retrieve full text articles and when clarification regarding inclusion criteria e.g. participant age, is required.

Study selection criteria

Inclusion criteria are empirical studies conducted in LMICs, as defined by the Organisation for Economic Co-operation and Development [31], irrespective of the study design, whose focus is on the knowledge, attitudes or experiences of stakeholders towards self-harm and/or suicide, where participants are aged 16 years and above. Studies that include stakeholders' knowledge, attitudes and

For peer review only - http://bmjopen.bmj.com/site/about/guidelines.xhtml

experiences of suicide and self-harm related to those under 16 will be included. Stakeholders are people who have experienced self-harm and/or have attempted suicide themselves, relatives, friends, co-workers, and healthcare workers of those who have self-harmed, attempted or completed suicide and people in the social, healthcare, government, and criminal justice sectors. Exclusion criteria are studies conducted in high income countries (HICs) and studies whose participants are not aged 16 years and above. Studies whose main focus is on the prevalence and/or predictors of self-harm and/or suicide, relationships between state and/or trait characteristics and self-harm and/or suicide, euthanasia, terrorism, or epidemiology will also be excluded. Systematic and literature reviews will be consulted for relevant references but will not be included in the review. Opinion pieces, editorials, book reviews, and conference and poster abstracts will not be included in the review.

The selection of studies for inclusion will adhere to the Cochrane guidelines and the process of selection of eligible studies will be illustrated via a PRISMA diagram [29]. Following deduplication of search results in EndNote, the following screening process will be undertaken in order to select studies for inclusion in the systematic review:

1) Titles and abstracts will be read by two reviewers independently, and relevance and fit with the inclusion criteria will be assessed. Those of no obvious relevance will be excluded and any disagreements will be resolved with a third reviewer (and the wider expert group if necessary).

2) Full text articles of remaining studies will be retrieved and read by two reviewers independently to assess their suitability for inclusion in the final review, disagreements will be resolved by discussion with a third reviewer (and the wider expert group if necessary). Both reviewers will populate a piloted pro-forma for each full text paper read (see Appendix 2).

For peer review only - http://bmjopen.bmj.com/site/about/guidelines.xhtml

Data extraction

Data will be extracted from selected studies by one reviewer, and a second reviewer will check for accuracy. Extracted data will be recorded on a piloted pro-forma (see Appendix 2), and will reflect the inclusion criteria and the designated aims of the review, derived from the article as a whole. Discrepancies will be resolved through discussion (with the wider expert group if necessary). Additional data will be requested from study authors when necessary. Data extraction of qualitative studies (and for qualitative components in studies with mixed methods) will adhere to the same methods and will be reviewed independently.

Outcomes

Outcomes of interest include:

- The identification of relevant information on stakeholders' knowledge, attitudes and experiences of self-harm and suicide, particularly in South Asia and in countries with comparable healthcare systems and cultural backgrounds
- The quantitative methods and measures that have been used to investigate stakeholders' attitudes towards and knowledge about self-harm and suicide and their psychometric properties
- The qualitative methods that have been used to investigate stakeholders' attitudes towards, knowledge about, and experiences of self-harm and suicide.

The identified outcomes will inform the development of a survey on knowledge, attitudes and well-being in South Asia as part of the SASHI project.

Quality assessment

All eligible studies will be subject to quality appraisal. The quality of included quantitative studies will be appraised using the STROBE checklist [32]. The STROBE Statement consists of a checklist of 22 items, which relate to the title, abstract, introduction, methods, results and discussion sections of articles. Eighteen items are common to cohort studies, case-control studies and cross-sectional studies

For peer review only - http://bmjopen.bmj.com/site/about/guidelines.xhtml

and four are specific to each of the three study designs. The quality of included qualitative studies will be appraised using the CASP checklist [33]. The 10-item CASP tool was considered to be the most suitable tool to consider the quality parameters of qualitative work, and is a well-validated and accepted tool [28]. Both the STROBE and CASP checklists will be applied independently by two reviewers and any disagreements will be resolved with a third reviewer (and the wider expert group if necessary).

Studies will not be excluded on the basis of poor quality alone, rather all studies that meet the inclusion criteria will be included in the review. This low threshold for inclusion will be applied so that the review can benefit from researcher insight and theoretical as well as empirical contributions. The relative quality of included studies will be critically considered and discussed in the review.

Descriptive analysis and data synthesis

We anticipate that the quantitative studies included in the review will be heterogenous and this will prevent meta-analysis. We will provide a narrative synthesis of quantitative studies, structured around population characteristics and the geographical region of studies. We will provide summaries of the quantitative methods and measures used to investigate stakeholders' attitudes towards and knowledge about self-harm and suicide and their psychometric properties.

Meta-ethnography will be used to synthesise qualitative studies [34]. Initially reciprocal translation will be performed by comparing the concepts presented in different studies. A chronological approach will be taken to reciprocal translation; studies will be arranged chronologically, concepts from papers one and two will be compared, and the synthesis of papers one and two will then be compared with paper three, and so forth, as is described elsewhere [35]. When contradictions between studies are identified, we will perform refutational synthesis by exploring and explaining these. A 'lines-of-argument' synthesis, that links and explains concepts presented by different studies, will be conducted so that an interpretation of all included studies can be presented.

For peer review only

For peer review only - http://bmjopen.bmj.com/site/about/guidelines.xhtml

Two reviewers will lead data synthesis. Emergent analysis, and any discrepancies, will be discussed with other members of the review team. Microsoft Office software will be used to facilitate data synthesis.

Patient and public involvement

No patients or members of the public were involved in the design of this study.

Amendments

An amendment has been made to the initial registration of this systematic review in PROSPERO, which originally stated that studies from both HICs and LMICs would be included in the review. The PROSPERO record was amended to state that only studies from LMICs will be included in this review, and studies from HICs will be excluded from this review. Any further amendments to this protocol will be documented in the full review.

ETHICS AND DISSEMINATION

Ethics approval is not required as this is a protocol for the systematic review of previously published data. In addition to a report to the funding body, we intend to submit the systematic review for publication in a high-impact, peer-reviewed journal. We will select an open access journal to ensure free access to undergraduate and graduate students, researchers, academics and research groups. Results will also be disseminated at conferences seminars, congresses and symposia and to relevant stakeholders.

Acknowledgements: The authors would like to thank Mrs Nia Morris (NM) from BCUHB library for her contribution to the development of the search strategy.

Contributions: CR, RM, PH, AK, MK conceived the research idea. SN led the development of the search strategy, with input from NM, RM, AK, PH. RM, SN, MK, AK, PH, CR conceived the

For peer review only

For peer review only - http://bmjopen.bmj.com/site/about/guidelines.xhtml

manuscript. RM lead the drafting of the manuscript with contributions from SN, AE, SB, MK, AK, PH, NC, CR. All authors approved the final manuscript.

Funding: This manuscript was funded by UK Research and Innovation Global Challenge Research Fund (ref: MR/P028144/1). The finder did not have any role in developing this protocol.

Competing interests: None declared

References

[1] World Health Organization. Preventing suicide: a global imperative. World Health Organization, 2014. Available: https://apps.who.int/iris/bitstream/handle/10665/131056/9789241564779_eng.pdf;jsessionid=F6255CAAD6496DA078DE41FAB0C5CCBF?sequence=1 . [Accessed 04 May 2020].

[2] Jordans MJD, Kaufman A, Brenman NF, *et al*. Suicide in South Asia: a scoping review. *BMC Psychiatry* 2014;**14**:358.doi:10.1186/s12888-014-0358-9 pmid: https://www.ncbi.nlm.nih.gov/pubmed/25539951

[3] United Nations. Department of economic and social affairs sustainable development: Ensure healthy lives and promote well-being for all at all ages. United Nations, 2015. Available: https://sdgs.un.org/goals/goal3 [Accessed 20 December 2020]

[4] Ward E, McCartney T, Arscott-Mills S, *et al*. The Jamaica injury surveillance system: a profile of the intentional and unintentional injuries in Jamaican hospitals. *West Indian Med J* 2010;**59**:7-13. pmid: https://www.ncbi.nlm.nih.gov/pubmed/20931906

For peer review only - http://bmjopen.bmj.com/site/about/guidelines.xhtml

[5] World Health Organization. Suicide in the World: global health estimates. World Health Organization, 2019. Available: https://www.who.int/publications/i/item/suicide-in-the-world [Accessed 20 December 2020]

[6] Aggarwal S. Suicide in India. *Br Med Bull* 2015;**114**:127-134.doi:10.1093/bmb/ldv018 pmid: https://www.ncbi.nlm.nih.gov/pubmed/25958380

[7] Gururaj G, Isaac MK, Subbakrishna DK, *et al*. Risk factors for completed suicides: a case-control study from Bangalore, India. *Inj Control Saf Promot* 2004;**3**:183-191.doi:10.1080/156609704/233/289706 pmid: https://www.ncbi.nlm.nih.gov/pubmed/15764105

[8] Laloe V. Patterns of deliberate self-burning in various parts of the world: a review. *Burns* 2004;**30**:207-215.doi:10.1016/j.burns.2003.10.018 pmid: https://www.ncbi.nlm.nih.gov/pubmed/15082345

[9] Vijayakumar L. Suicide in women. *Indian J Psychiatry* 2015;**57**:S233-S238.doi:10.4103/0019-5545.161484 pmid: https://www.ncbi.nlm.nih.gov/pubmed/26330640

[10] Jacobson CM, Gould M. The epidemiology and phenomenology of non-suicidal self-injurious behavior among adolescents: a critical review of the literature. *Arch Suicide Res* 2007;**11**:12-47doi:10.1080/13811110701247602 pmid: https://www.ncbi.nlm.nih.gov/pubmed/17453692

[11] Muehlenkamp JJ. Self-injurious behavior as a separate clinical syndrome. *Am J Orthopsychiatry* 2005;**75**:324-333.doi:10.1037/0002-9432.75.2.324 pmid: https://www.ncbi.nlm.nih.gov/pubmed/15839768

For peer review only

For peer review only - http://bmjopen.bmj.com/site/about/guidelines.xhtml

[12] Nock MK. Self-injury. *Annu Rev Clin Psychol* 2010;**6**:339-363 doi:10.1146/annurev.clinpsy.121208.131258. pmid: https://www.ncbi.nlm.nih.gov/pubmed/20192787

[13] Walsh BW. *Treating self-injury: A practical guide* . New York, USA: Guilford Press 2005

[14] Gholamrezaei M, Stefano JD, Heath NL. Nonsuicidal self-injury across cultures and ethnic and racial minorities: a review. *Int J Psychol* 2017;**52**:316-326. doi:10.1002/ijop.12230 pmid: https://www.ncbi.nlm.nih.gov/pubmed/26644040

[15] Aggerwal S, Berk M. Nonsuicidal self-injury in Indian adolescents: nonexistent or unacknowledged? *Int J Soc Psychiatry* 2015;**61**:516-517doi:10.1177/0020764015579739 pmid: https://www.ncbi.nlm.nih.gov/pubmed/26209701

[16] Aggarwal S, Patton G, Reavley N. Youth self-harm in low- and middle- income countries: systematic review of the risk and protective factors. *Int J Soc Psychiatry* 2017;**63**:359-375doi: 10.1177/0020764017700175 pmid: https://www.ncbi.nlm.nih.gov/pubmed/ 28351292

[17] Hamza CA, Stewart SL, Willoughby T. Examining the link between nonsuicidal self-injury and suicidal behaviour: a review of the literature and integrated model. *Clin Psychol Rev* 2012;**6** 482-95.doi:10.1016/j.cpr.2012.05.003. pmid: https://www.ncbi.nlm.nih.gov/pubmed/22717336

[18] Owens D, Horrocks J, House A. Fatal and non-fatal repetition of self-harm: systematic review. *Br J Psychiatry* 2002;**181**:193-99. doi: 10.1192/bjp.181.3.193 pmid: https://www.ncbi.nlm.nih.gov/pubmed/12204922

For peer review only - http://bmjopen.bmj.com/site/about/guidelines.xhtml

[19] Hawton K, Sanders KEA, O'Connor RC. Self-harm and suicide in adolescents. *Lancet* 2012;**379**:2373-82. doi: 10.1016/S0140-6736(12)60322-5 pmid: https://www.ncbi.nlm.nih.gov/pubmed/22726518

[20] Yip PSF, Caine E, Yousef S, *et al*. Means restriction for suicide prevention. *Lancet* 2012;379:2393-2399. doi: 10.1016/S0140-6736(12)60521-2

[21] World Health Organization. National suicide prevention strategies: progress, examples and indicators. World Health Organization, 2018. Available: https://apps.who.int/iris/bitstream/handle/10665/279765/9789241515016-eng.pdf?ua=1 . [accessed 04 May 2020].

[22] Karman P, Kool N, Poslawsky IE, *et al.* Nurses' attitudes towards self-harm: a literature review. *J Psychiatr Ment Health Nurs* 2015;**22**:65-75. doi: 10.1111/jpm.12171 pmid: https://www.ncbi.nlm.nih.gov/pubmed/25490929

[23] O'Connor S, Glover L. Hospital staff experiences of their relationships with adults who self-harm: a meta-synthesis. *Psychol Psychother* 2017;**90**:480-501. doi: 10.1111/papt.12113. pmid: https://www.ncbi.nlm.nih.gov/pubmed/28035740

[24] Rees N, Rapport F, Thomas G, *et al*. Perceptions of paramedic and emergency care workers of those who self-harm: a systematic review of the quantitative literature. *J Psychosom Res* 2014:**77**;449-456. doi: 10.1016/j.jpsychores.2014.09.006. pmid: https://www.ncbi.nlm.nih.gov/pubmed/25263398

[25] Saunders KEA, Hawton K, Fortune S, *et al*. Attitudes and knowledge of clinical staff regarding people who self-harm: a systematic review. *J Affect Disord* 2012;**139**:205-216. doi: 10.1016/j.jad.2011.08.024 pmid: https://www.ncbi.nlm.nih.gov/pubmed/21925740

For peer review only - http://bmjopen.bmj.com/site/about/guidelines.xhtml

[26] Booth A. Clear and present questions: formulating questions for evidence based practice. *Library Hi Tech* 2006;**24**:355-368. doi: 10.1108/07378830610692127

[27] Shamseer L, Moher D, Clarke M, *et al*. Preferred reporting items for systematic review and meta-analysis protocols (PRISMA-P) 2015: elaboration and explanation. *BMJ* 2015;**350**:g7647. doi: 10.1136/bmj.g7647 pmid: https://www.ncbi.nlm.nih.gov/pubmed/25555855

[28] Higgins JPT, Green S (editors). Cochrane handbook for systematic reviews of interventions Version 6. The Cochrane Collaboration, 2019. Available: http://handbook.cochrane.org. [Accessed 04 May 2020].

[29] Liberati A, Altman DG, Tetzlaff J, *et al*. The PRISAM statement for reporting systematic reviews and meta-analyses of studies that evaluate healthcare interventions: explanation and elaboration. *BMJ* 2009;**339**:b2700. doi: 10.1136/bmj.b2700 pmid: https://www.ncbi.nlm.nih.gov/pubmed/19622552

[30] McPhillips R, Nafees S, Krishna M, *et al*. Knowledge, attitudes, and experiences of self-harm and suicide in low and middle income countries: a systematic review. PROSPERO 2019 CRD42019135323. Available: https://www.crd.york.ac.uk/prospero/display_record.php?ID=CRD42019135323 [Accessed 04 May 2020]

[31] Organisation for Economic Co-operation and Development. DAC List of ODA recipients for reporting on aid in 2018 and 2019. Organisation for Economic Co-operation and Development, 2019. Available: http://www.oecd.org/dac/financing-sustainable-development/development-finance-standards/daclist.htm [Accessed 04 May 2020]

For peer review only - http://bmjopen.bmj.com/site/about/guidelines.xhtml

For peer review only

[32] von Elm E, Altman DG, Egger M, *et al.* STROBE Initiative. The strengthening the reporting in observational studies in epidemiology (STROBE) statement guidelines for reporting observational studies. Lancet 2007;**370**:1453-57 doi: 0.1016/S0140-6736(07)61602-X pmid: https://www.ncbi.nlm.nih.gov/pubmed/18064739

[33] Critical Appraisal Skills Programme. CASP qualitative checklist. Critical Appraisal Skills Programme , 2018. Available: https://casp-uk.net/wp-content/uploads/2018/01/CASP-Qualitative-Checklist-2018.pdf
[Accessed: 04 May 2020]

[34Noblit GW, Hare RD. Meta-ethnography: synthesising qualitative studies. Sage Publications.

[35] Atkins S, Lewin S, Smith H *et al*. Conducting a meta-ethnography of qualitative literature: lessons learnt. *BMC Med Res Methodol* 2008;**8**:21. doi: 10.1186/1471-2288-8-21 pmid: https://www.ncbi.nlm.nih.gov/pubmed/ 18416812

For peer review only - http://bmjopen.bmj.com/site/about/guidelines.xhtml

For peer review only

For peer review only - http://bmjopen.bmj.com/site/about/guidelines.xhtml

Supplementary file 1: PRISMA-P (Preferred Reporting Items for Systematic review and Meta-Analysis Protocols) 2015 checklist: recommended items to address in a systematic review protocol*

Section and topic	Item No	Checklist item	Complete
**ADMINISTRATIVE INFORMATION**			
Title:			
Identification	1a	Identify the report as a protocol of a systematic review	X
Update	1b	If the protocol is for an update of a previous systematic review, identify as such	N/A
Registration	2	If registered, provide the name of the registry (such as PROSPERO) and registration number	X
Authors:			
Contact	3a	Provide name, institutional affiliation, e-mail address of all protocol authors; provide physical mailing address of corresponding author	X
Contributions	3b	Describe contributions of protocol authors and identify the guarantor of the review	X
Amendments	4	If the protocol represents an amendment of a previously completed or published protocol, identify as such and list changes; otherwise, state plan for documenting important protocol amendments	X
Support:			
Sources	5a	Indicate sources of financial or other support for the review	X
Sponsor	5b	Provide name for the review funder and/or sponsor	X
Role of sponsor or funder	5c	Describe roles of funder(s), sponsor(s), and/or institution(s), if any, in developing the protocol	X
**INTRODUCTION**			
Rationale	6	Describe the rationale for the review in the context of what is already known	X
Objectives	7	Provide an explicit statement of the question(s) the review will address with reference to participants, interventions, comparators, and outcomes (PICO)	X
**METHODS**			
Eligibility criteria	8	Specify the study characteristics (such as PICO, study design, setting, time frame) and report characteristics (such as years considered, language, publication	X

For peer review only

		status) to be used as criteria for eligibility for the review	
Information sources	9	Describe all intended information sources (such as electronic databases, contact with study authors, trial registers or other grey literature sources) with planned dates of coverage	X
Search strategy	10	Present draft of search strategy to be used for at least one electronic database, including planned limits, such that it could be repeated	X
Study records:			
Data management	11a	Describe the mechanism(s) that will be used to manage records and data throughout the review	X
Selection process	11b	State the process that will be used for selecting studies (such as two independent reviewers) through each phase of the review (that is, screening, eligibility and inclusion in meta-analysis)	X
Data collection process	11c	Describe planned method of extracting data from reports (such as piloting forms, done independently, in duplicate), any processes for obtaining and confirming data from investigator	X
Data items	12	List and define all variables for which data will be sought (such as PICO items, funding sources), any pre-planned data assumptions and simplifications	X
Outcomes and prioritization	13	List and define all outcomes for which data will be sought, including prioritization of main and additional outcomes, with rationale	X
Risk of bias in individual studies	14	Describe anticipated methods for assessing risk of bias of individual studies, including whether this will be done at the outcome or study level, or both; state how this information will be used in data synthesis	X
Data synthesis	15a	Describe criteria under which study data will be quantitatively synthesised	X
	15b	If data are appropriate for quantitative synthesis, describe planned summary measures, methods of handling data and methods of combining data from studies, including any planned exploration of consistency (such as I^2, Kendall's τ)	N/A
	15c	Describe any proposed additional analyses (such as sensitivity or subgroup analyses, meta-regression)	X
	15d	If quantitative synthesis is not appropriate, describe the type of summary planned	X
Meta-bias(es)	16	Specify any planned assessment of meta-bias(es) (such as publication bias across studies, selective reporting within studies)	N/A
Confidence in cumulative evidence	17	Describe how the strength of the body of evidence will be assessed (such as GRADE)	N/A

For peer review only

*** It is strongly recommended that this checklist be read in conjunction with the PRISMA-P Explanation and Elaboration (cite when available) for important clarification on the items. Amendments to a review protocol should be tracked and dated. The copyright for PRISMA-P (including checklist) is held by the PRISMA-P Group and is distributed under a Creative Commons Attribution Licence 4.0.**

From: Shamseer L, Moher D, Clarke M, Ghersi D, Liberati A, Petticrew M, Shekelle P, Stewart L, PRISMA-P Group. Preferred reporting items for systematic review and meta-analysis protocols (PRISMA-P) 2015: elaboration and explanation. BMJ. 2015 Jan 2;349(jan02 1):g7647.

For peer review only

Appendix 1: Knowledge, attitudes, and experiences of self-harm and suicide in low and middle income countries: Medline search strategy

Self-harm and suicide terms

1 Suicide/
2 Suicide.mp
3 Suicide, Attempted/
4 Suicide, Attempted.mp
5 Self-Injurious Behavior/
6 Self-Injurious Behavior.mp
7 Self-Mutilation/
8 Self-Mutilation.mp
9 Suicidal Ideation/
10 Suicidal Ideation.mp
11 Attempted Suicide/
12 Attempted Suicide.mp
13 Drug Overdose/
14 Drug Overdose.mp
15 ((Self adj2 cut$) or (para adj suicid$) or (attempt$ adj suicide$) or (suicid$ adj behavio$)).mp.
16 (auto?mutilat$ or cutt$ or over?dos$ or self?destruct$ or self?harm$ or self?immolat$ or self?inflict$ or self?injur$ or self?mutilat$ or self?poison$ or suicide$ or self?burn$).mp.
17 Or/1-16

Stakeholder terms

18 exp Health Personnel/
19 Health Personnel.mp
20 exp PHYSICIANS/
21 PHYSICIANS.mp
22 exp Personnel, Hospital/
23 Personnel, Hospital.mp
24 Social Workers/
25 Social Workers.mp
26 ((doctor$ or physician$ or hosp$ or medic$ or health?care or social or wel?fare) adj (profession$ or staff$ or officer$ or person$ or worker$)).mp
27 Family/
28 Famil$.mp
29 Friends/
30 Friend$.mp
31 Caregivers/
32 Caregiver$.mp
33 Criminals/
34 Criminal$.mp
35 Prisoners/
36 Prison$.mp
37 Social Justice/
38 Soci$ adj Just$.mp
39 (partner$ or parent$ or grandparent$ or children or sibling$ or famil$ or friend$ or relative$).mp.
40 (communit$ or societ$ or government$).mp.
41 (criminal$ or offender$ or prisoner$ or in?mate$).mp.
42 (justice adj system$).mp.

For peer review only

43 or/18-42

Knowledge and attitude terms

44 Attitude/
45 Attitude of Health Personnel/
46 Attitude to Death/
47 Knowledge/
48 Health Knowledge, Attitudes, Practice/
49 Awareness/
50 Education/
51 Health Education/
52 ((train$ or trainer$ or experienc$ or perspectiv$ or knowledg$ or attitud$ or awaren$ or educat$) adj health).mp.
53 social behavior/ or help-seeking behavior/ or self-control/ or shyness/ or social adjustment/ or social isolation/ or social marginalization/ or social skills/ or social stigma/ or social exclusion/ or social inclusion
54 Prejudice/
55 Taboo/
56 exp Shame/
57 (tabo$ or sham$ or stigma$ or prejudice$ or help?seek$ behavior$ or self?control$ or shy$ or (social adj adjust$ or behavio$ or isolate$ or margin$ or Stigm$ or skil$)).mp
58 or/44-57

LMIC terms

59 Developing Countries/
60 "low and middle income countr$".ab,ti.
61 LMIC.mp.
62 india/ or sikkim/ or pakistan/
63 exp Asia/
64 or/59-63

Self-harm and suicide, stakeholders, knowledge and attitude and LMIC combined (with limits)

65 17 and 43 and 58 and 64
66 limit 65 to humans
67 limit 66 (adolescent <13 to 17 years> or adult <18 to 64 years> or aged <65+ years>) (aged or "aged, 80 and over" or "frail elderly" or "middle aged" or "young adult" or adults or "teenager" or adolescent).mp

For peer review only

For peer review only - http://bmjopen.bmj.com/site/about/guidelines.xhtml

Appendix 2: Knowledge, attitudes, and experiences of self-harm and suicide in low and middle income countries systematic review: data extraction form

Notes for reviewers

Record any missing information as unclear or not described to ensure it is clear that the information was not found in the paper, not that you forgot to extract it

**General information**		
Date form completed		
Reviewer extracting data		
Study title		
Study authors		
Journal		
Year of publication		
Study author contact details		
Notes		
**Study eligibility for inclusion in review**		
Main focus on stakeholders' knowledge, attitudes and experiences of self-harm and/or suicide (excluding euthanasia or terrorism)	*Yes/No*	*Location in text (pg. #)*
Study population aged 16 and above (or can data from those aged 16 and above only can be extracted?)	*Yes/No*	*Location in text (pg. #)*
Low-middle income country	*Yes/No*	*Location in text (pg. #)*
Include or exclude	*Include/Exclude*	
Reason for exclusion	*Reasons for exclusion*<br>*1) Knowledge, attitudes and experience of self-harm/suicide not main concern of study (including terrorism and euthanasia) include main phenomenon being studied in notes – to be reviewed after 25 studies*<br>*1a) Completely irrelevant topic e.g. paper on depression, no mention of self-harm/suicide 1b) Focus on prevalence of suicide/self-harm*<br>*1c) Focus on risk factors of suicide/self-harm*<br>*1d) Focus on intervention only*<br>*1e) Mention of self-harm/suicide however topic not relevant to attitudes, knowledge and experiences of self-harm/suicide*<br>*2) Research not conducted in LMICs*<br>*3) Research population not 16 and over*<br>*4) Literature review*<br>*5) Commentary, book review, editorial*	
Notes		
**Characteristics of included studies: Participants**		

For peer review only

	Description as stated in paper	*Location in text (pg. #)*
Study location (Country and state/city/area) e.g. India, Bangalore		
Study setting e.g. hospital, community		
Study population e.g. nurses, community members		
Informed consent obtained	*Yes, No, Unclear*	
Total number of participants		
Exclusions and withdrawals		
Participant demographics e.g.<br>Age<br>Sex<br>Race/Ethnicity<br>Religious beliefs<br>Mental illness diagnosis<br>Physical illness diagnosis<br>Other demographics		
Notes		
**Characteristics of included studies: Methods**		
	Description as stated in paper	*Location in text (pg. #)*
Aim of study/Research question(s) (implicit or explicit in text?)		
Study methodology (or methodologies)		
Quantitative measures used e.g. Acceptability of Suicide Scale and information on whether measure is validated (if applicable)		
Quantitative analysis methods and procedure		
Qualitative methods used e.g. focus group, one-to-one interviews, vignettes (if applicable)		
Theoretical/epistemological perspectives underpinning qualitative research (explicit or reviewer's interpretation)		
Qualitative data analysis methods and procedure		
Start date and end date		
Notes		
**Characteristics of included studies: Results**		
	Description as stated in paper	*Location in text (pg. #)*
Qualitative results – direct quotes from participants (first order)		

For peer review only

Qualitative results – study author's interpretations of data (second order)		
Quantitative results		
Indicators of acceptability to users (if applicable)		
Suggested mechanisms of intervention action (if applicable)		
**Characteristics of included studies: Other information**		
	Description as stated in paper	*Location in text (pg. #)*
Key conclusions of authors		
References to other relevant studies		
Correspondence required by reviewers for further information (who, when, what requested)		
Notes		

For peer review only
